# Susceptibility of *Plasmodium falciparum* to artemisinins and *Plasmodium vivax* to chloroquine in Phuoc Chien Commune, Ninh Thuan Province, south-central Vietnam

**DOI:** 10.1186/s12936-019-2640-2

**Published:** 2019-01-17

**Authors:** Nguyen Chinh Phong, Marina Chavchich, Huynh Hong Quang, Nguyen Ngoc San, Geoffrey W. Birrell, Ilin Chuang, Nicholas J. Martin, Nguyen Duc Manh, Michael D. Edstein

**Affiliations:** 1 Vietnam People’s Army Military Institute of Preventive Medicine, Hanoi, Vietnam; 2 Australian Defence Force Malaria and Infectious Disease Institute, Brisbane, Australia; 3 Institute of Malariology, Parasitology and Entomology, Quy Nhon, Vietnam; 4 Naval Medical Research Center, Silver Spring, USA; 5 Naval Medical Research Unit-TWO, Singapore, Singapore

**Keywords:** Malaria, Vietnam, *Plasmodium falciparum*, *Plasmodium vivax*, Artesunate, Artemether, Chloroquine, Dihydroartemisinin, Lumefantrine, Piperaquine, *Pfkelch*-13, In vitro drug susceptibility testing

## Abstract

**Background:**

Reduced artemisinin susceptibility and artemisinin-based combination therapy (ACT)-resistance against *Plasmodium falciparum* and chloroquine (CQ)-resistant *P. vivax* malaria has been reported in Vietnam. Two therapeutic efficacy studies were conducted in Thuan Bac District (Ninh Thuan Province, Vietnam) in 2015 and 2016 to determine the extent of reduced artemisinin susceptibility and ACT resistant falciparum malaria, and CQ-resistant vivax malaria were present.

**Methods:**

Twenty-seven patients with falciparum malaria were randomized to receive artesunate alone (AS ~ 4 mg/kg/day) for 4 days followed by dihydroartemisinin (DHA) (2.2 mg/kg)–piperaquine (PPQ) (18 mg/kg) daily for 3 days or artemether (AM) (1.7 mg/kg)–lumefantrine (LUM) (12 mg/kg) twice daily for 3 days. Sixteen subjects with vivax malaria received CQ (total 25 mg/kg over 3 days). The therapeutic efficacy study for treating falciparum malaria was complemented with molecular analysis for artemisinin and piperaquine resistance, and in vitro drug susceptibility testing. Patient’s drug exposure following both falciparum and vivax treatment studies was determined.

**Results:**

Twenty-five of 27 patients treated with the artemisinin regimens completed the 42-day follow-up period. None had parasites present on day 3 after commencing treatment with no incidence of recrudescence (100% curative rate). One patient on AS + DHA–PPQ was lost to follow-up and one patient had *Plasmodium falciparum* and *Plasmodium vivax* infection on day 0 by PCR. Of the vivax patients, 15 of 16 completed CQ treatment and two had a recurrence of vivax malaria on day 28, a failure rate of 13.3% (2/15). No mutations in the *Pfkelch*-13 gene for artemisinin resistance or *exo*-*E415G* gene polymorphism and amplification in *plasmepsins 2* and *3* for piperaquine resistance were observed. In vitro testing of patient’s falciparum parasites indicated susceptibility (low IC_50_ nM values) to dihydroartemisinin, lumefantrine, piperaquine and pyronaridine. Patient’s drug exposure to artesunate and lumefantrine was comparable to published data, however, blood CQ concentrations were lower.

**Conclusions:**

Clinical findings, molecular analysis and in vitro testing revealed that the falciparum parasites at Phuoc Chien Commune were artemisinin susceptible. The clinical failure rate of the 15 vivax patients who completed CQ treatment was 13%. Further studies are required to determine whether CQ-resistant vivax malaria is present at the commune.

**Electronic supplementary material:**

The online version of this article (10.1186/s12936-019-2640-2) contains supplementary material, which is available to authorized users.

## Background

Although the number of malaria cases has been declining over the past decade worldwide, malaria is still a critical infectious disease causing more than 200 million malaria cases and approximately 450,000 deaths annually [1]. Control of *Plasmodium falciparum* malaria is made more difficult by the ongoing spread of anti-malarial drug resistance to standard drugs. Since 2006, Cambodia has experienced the emergence of artesunate (AS), artesunate–mefloquine (AS–MQ) and dihydroartemisinin–piperaquine (DHA–PPQ) resistance [2–5]. In neighbouring Vietnam, there is now emerging evidence of reduced susceptibility of artemisinins with indications of DHA–PPQ resistance in Binh Phuoc Province, recognized in 2015 [6]. Therapeutic efficacy studies of first-line treatment with DHA–PPQ at national sentinel sites has seen a delay in parasite clearance in Gia Lai Province (2010), Dak Nong Province (2011), Quang Nam Province (2012), Khanh Hoa Province (2014) and Ninh Thuan Province (2015) with over 10% of patients microscopically positive on day 3 after starting treatment [6]. It is crucial to know whether reduced artemisinin susceptibility and DHA–PPQ resistance has spread elsewhere in Vietnam and to establish more effective artemisinin-based combination therapy (ACT).

In 2012 and 2013, DHA–PPQ was still highly effective in Vietnam (> 98% cure rate), but by 2014 the cure rate of DHA–PPQ had declined to 93% in Binh Phuoc Province [7]. This finding highlights the need to seek out alternative artemisinin-based combinations in case the efficacy of DHA–PPQ continues to decline throughout Vietnam. As a potential alternative to DHA–PPQ, an ACT that is commonly used in Africa is artemether–lumefantrine (AM–LUM) (marketed as Coartem^®^). In 2001, the National Institute of Malariology, Parasitology and Entomology (Hanoi, Vietnam) reported a cure rate of 100%, with a 28-day follow-up period in 40 adult patients treated with AM–LUM for *P. falciparum* from Binh Phuoc Province [Nong Thi Tien and Tran Quan Binh, unpublished data]. Other than this study, there is little published information on the efficacy of Coartem^®^ in Vietnam, an ACT recommended by the WHO for the treatment of uncomplicated *P. falciparum* in young children and adults worldwide.

For many years, researches have focused on malignant *P. falciparum* because of drug resistance, but pernicious *P. vivax* malaria is also a highly debilitating disease and is a major cause of morbidity [8]. In Southeast Asia and South America vivax malaria accounts for up to 70% of malaria cases [9]. Although chloroquine (CQ) still remains an effective first-line treatment of *P. vivax* malaria in many countries, including Vietnam, CQ resistance is emerging worldwide and severe forms of the disease pose a significant global health threat [9]. In *P. vivax* endemic areas, relapse of the parasite is also an important source of malaria transmission. Where there is a high treatment failure rate with CQ (> 10%), the WHO [1] encourages countries to change their first-line treatment to an ACT.

In Vietnam, vivax malaria is prevalent in the north of the country as well as along the western border. In a therapeutic efficacy study of CQ for the treatment of *P. vivax* conducted from 1997 to 2000 in Binh Thuan Province in south Vietnam, CQ-resistant vivax malaria was reported in 18/113 (16%) patients with a 28-day follow-up period, but blood CQ concentrations were not measured thus adequate drug exposure to CQ could not be confirmed [10]. A study conducted in 2009 to 2011 in Quang Nam Province in central Vietnam revealed CQ-resistant in *P. vivax* malaria. In this study, three patients with recurrent *P. vivax* malaria were determined by CQ blood concentrations to have had adequate drug exposure [11]. However, a more recent study in Binh Phuoc Province conducted in 2013 and 2014 reported CQ to be highly efficacious for the treatment of *P. vivax* malaria in southern Vietnam where no recurrence of vivax infections were detected over the first 28 days of follow-up [12]. As with other countries in Southeast Asia, Vietnam is confronted with the potential spread of CQ-resistant vivax malaria. However, the prevalence of CQ-resistant vivax malaria in Vietnam is still unknown.

The findings from two therapeutic efficacy studies at a commune in Thuan Bac District in Ninh Thuan Province, south-central Vietnam are reported in this study. The objectives were to determine whether reduced AS susceptibility, DHA–PPQ resistance or AM–LUM resistance were present in treating uncomplicated *P. falciparum* malaria and whether CQ-resistant *P. vivax* malaria was also present. In support of the clinical studies, molecular analysis and in vitro drug susceptibility testing of *P. falciparum* parasites were conducted as well as measuring the patient’s blood or plasma drug concentrations to ascertain the level of drug exposure.

## Methods

### Study site and patients

The therapeutic efficacy studies were conducted from May 2015 to December 2016 at Phuoc Chien Commune (Thuan Bac District, Ninh Thuan Province, Additional file 1: Figure S1. Map of Phuoc Chien Commune) in south-central Vietnam. The commune located near the coast, about 270 km north of Ho Chi Minh City, has a population of about 4000 people, with most belonging to the Raglai ethnic group. Malaria transmission is low and occurs perennially with two peaks (May–June and October–November) [13]. *Anopheles dirus* and *Anopheles maculatus* are the main malaria vectors on the forested hill areas near the commune [14].

Subjects presenting with malaria symptoms, such as fever, rigors/chills, headache and fatigue, at the Phuoc Chien Commune health station were screened for malaria infection. Patients who were diagnosed with uncomplicated *P. falciparum* malaria by blood film microscopy were invited to participate in the AS + DHA–PPQ and AM–LUM treatment study if they met the following inclusion criteria: age 5–65 years; a blood slide confirmed *P. falciparum* monoinfection with a parasite density of 500–200,000 asexual parasites/µL; tympanic temperature ≥ 38 °C at the time of enrolment or history of fever during the preceding 24 h and willingness to be followed-up for 42 days after starting treatment. Exclusion criteria were as follows: patients with symptoms and/or signs of severe malaria; evidence of another serious medical disease; a history of drug or alcohol abuse; anti-malarial treatment within the preceding 14 days; mixed *Plasmodium* species and positive pregnancy test for females ≥ 12 years old. With the exception of asexual parasitaemia between 200 and 100,000 parasites/μL and willingness to be followed-up for 28 days after starting CQ treatment; the inclusion and exclusion criteria for patients who were diagnosed with uncomplicated *P. vivax* malaria were the same as that for patients invited to participate in the *P. falciparum* malaria study.

A minimum sample size of 50 patients per arm was required for the study to be representative (n ≥ 50) [15], with the plan to enrol 70 patients with falciparum malaria and 40 patients with vivax malaria per year over 2 years. Written informed consent was obtained from each adult patient, from parents or legal guardians of enrolled children aged 8 to 14 years old and assent from children aged 8 to 14 years old.

The capture rate of malaria incidence in this study from the Phuoc Chien Commune health station was nearly 100%. Although there were some private clinics in the area, the law in Vietnam requires anyone with malaria must be referred to a Governmental commune health station or district hospital where treatment is free. Selling anti-malarial drugs is not allowed in Vietnam. Anti-malarial drugs are only available from public health facilities where malaria patients will receive treatment for free. Whenever a private medical facility diagnoses malaria the patient will be sent to a public health facility for treatment.

### Study design and drug treatments

In an open-label study, patients with falciparum malaria were randomized to two treatment groups: (AS ~ 4 mg/kg/day) for 4 days followed by a 3-day course of DHA (~ 2.2 mg/kg/day) plus PPQ (~ 18 mg/kg/day) and a 3-day course of AM (~ 1.7 mg/kg) plus LUM (~ 12 mg/kg) administered twice daily. Patients with vivax malaria were treated with CQ (10 mg/kg on days 0 and 1; 5 mg/kg of day 2). The drugs were administered under direct observation. AS (each tablet contained 50 mg of AS) and CQ diphosphate (each tablet contained 150 mg CQ base) were purchased from Mekophar Chemical Pharmaceutical Joint Stock Company (Ho Chi Minh City, Vietnam), DHA–PPQ marketed as Arterakine^®^ (each tablet contained 40 mg DHA and 320 mg PPQ as a tetraphosphate salt) was purchased from Central Pharmaceutical Factory No. 1 (Hanoi, Vietnam), AM–LUM marketed as Coartem^®^ (each tablet contained 20 mg of AM and 120 mg of LUM) was purchased from Novartis Pharmaceuticals (Lelystad, Netherlands) and primaquine diphosphate (each tablet contained 7.5 mg primaquine base) was purchased from Danapha (Da Nang, Vietnam). The drugs were given orally with at least 100 mL of water for AS, DHA–PPQ and CQ, and with milk containing at least 2 g of fat for AM–LUM, which is required to enhance drug absorption of the lipophilic LUM [16]. At the end of the 28-day follow-up period glucose-6-phosphate dehydrogenase normal patients treated with CQ received a full course of primaquine (a dose of 0.25 mg/kg/day × 14 days) to treat dormant liver stages (hypnozoites) of vivax malaria as per Vietnam Ministry of Health guidelines.

### Laboratory investigations and follow-up

Clinical assessment and parasite density were performed on days 0, 1, 2, 3, 7, 14, 21, and 28 for patients treated for either falciparum or vivax infections, with an additional two time points (days 35 and 42) for patients treated for falciparum malaria and on any day of recurrent malaria infection. Thick and thin blood films were collected before and at about every 12 h after commencement of treatment until blood films were negative for three consecutive examinations. The Giemsa-stained blood films were examined independently by two microscopists and parasitaemia was quantified by examination of 200 thick film fields (magnification 1000×) against 200 white blood cells (WBC), assuming a total WBC count of 6000/µL. When the number of asexual parasites was less than 10 per 200 WBC in follow-up blood films, counting was done against at least 500 WBC. A blood slide was considered negative when examination of 1000 WBC revealed no asexual parasites. Quality assurance of microscopy was performed by a WHO Level 1 malaria microscopist from ADFMIDI.

Parasite clearance time was the time in hours from starting treatment until the asexual parasite count fell below detectable levels in thick blood films. Patient’s tympanic body temperature was measured immediately before and at about every 12 h after starting treatment until their temperature was < 38 °C for two consecutive days. Fever clearance time was the time in hours from starting treatment until the patient’s tympanic body temperature remained < 38 °C for more than 48 h.

Blood spots on filter paper (3MM, Whatman) were collected before starting treatment (day 0), on day 7 and afterwards when blood films were obtained or when a recurrence of malaria occurred. Parasite DNA was extracted from filter paper blood spots using the QIAamp DNA mini kit (Qiagen Pty Ltd, Melbourne, Australia) and polymerase chain reaction (PCR) parasite genotyping was done to confirm *Plasmodium* species using a single round multiplex PCR for detection and identification of the four species, *P. falciparum*, *P. vivax*, *Plasmodium ovale* and *Plasmodium malariae* [17]. In order to differentiate reinfection from recrudescence, multiplex PCR analysis was performed on paired samples of *P. falciparum* DNA (day 0 and day of recurrence of parasitaemia) for allelic variation at three loci (merozoite surface proteins 1 and 2, and glutamate rich protein) [18]. If at least one allele of the recurrent parasitaemia was different to the before treatment parasitaemia, the infection was considered a reinfection.

### Patient tolerability to administered anti-malarials

Drug tolerability was assessed clinically. An adverse event was defined as any sign or symptom that occurred or became severe during the study regardless as to whether it was related to the medication. Adverse events were recorded by the study doctor at each drug administration as well as 24 h after the last dose. This post-dosing drug-tolerability assessment was conducted in order to better distinguish between disease effects and that of the contraindications of drug administration. Malaria infection, particularly during the acute phase can cause adverse effects such as nausea, abdominal pain, headache and dizziness, which can often be misattributable to drug adverse events [19]. In this study, no causal association was made between the adverse events and the administered drug.

### Molecular analysis for drug resistance

Markers for drug resistance were assessed in parasite DNA extracted from day 0 blood spots. A marker for artemisinin resistance in *kelch*-13 gene (from 427 to 687 amino acids) was sequenced in *P. falciparum* samples using nested PCR as previously described [20]. Polymorphism in exonuclease gene (*exo*-*E415G*) and copy number of *plasmepsins 2* and *3*, that have both been implicated in resistance to PPQ, were evaluated as previously described [21, 22].

### In vitro drug susceptibility testing of clinical isolates

Adult patients with falciparum malaria were invited to provide a blood sample before starting treatment for in vitro drug susceptibility testing of their isolates. The heparinized venous blood sample (2 mL) was centrifuged at 295×*g* for 10 min, the plasma was removed and remaining red blood cells were preserved by adding Glycerolyte57 solution (Baxter, Deerfield, IL, USA) and stored in liquid nitrogen at the field site. The cryopreserved parasites were transferred to the Australian Defence Force Malaria and Infectious Disease Institute (ADFMIDI) on dry-ice, where they were adapted to culture over a 2–3 week period [23]. For in vitro anti-malarial activity assessment, stocks of the following drugs: atovaquone (ATQ) was obtained from GlaxoSmithKline (Hertfordshire, UK); desethylamodiaquine (DAQ), dihydroartemisinin (DHA), piperaquine tetraphosphate (PPQ) were obtained from the WorldWide Antimalarial Resistance Network (WWARN QA/QC Reference Material Programme, Bangkok, Thailand), and chloroquine diphosphate (CQ), mefloquine hydrochloride (MQ), and pyronaridine tetraphosphate (PRN) were purchased from Sigma (St. Louis, MO, USA). The drugs were prepared to 1 mM in either dimethyl sulfoxide (DMSO), methanol or 50% methanol/50% water and subsequently diluted in culture media lacking hypoxanthine. Drug susceptibility of the field isolates was evaluated by measuring the inhibition of the radioactive [^3^H]-hypoxanthine uptake by parasites [24], with a 48 h incubation period as previously described [25]. The drug-sensitive D6 (originally from Sierra Leone) and the multidrug-resistant MRA1240 (Cambodian) lines of *P. falciparum* were run as reference strains. Drug IC_50_ values (i.e., concentrations that cause 50% inhibition of parasite growth or [^3^H]-hypoxanthine uptake, when compared with drug-free controls) were determined using a nonlinear regression analysis (GraphPad Prism V5.0, GraphPad Software, Inc., CA, USA).

### Ring-stage and piperaquine survival assays

The ring-stage survival assay (RSA) and PPQ survival assay (PSA) were carried out on all *P. falciparum* field isolates that were collected from patients prior to treatment with AS + DHA–PPQ as previously described [26, 27]. MRA1239^S^ (artemisinin-sensitive) and MRA1240^R^ (artemisinin-resistant) strains (Cambodia), obtained from BEI Resources (Manassas, VA, USA), were used as reference strains.

### Patient’s blood and plasma drug concentrations

For determining drug exposure in patients treated for falciparum malaria a blood sample (1 mL) was collected at 1 h after the last dose of AS and on day 7 after commencing AM–LUM treatment. Fluoride oxalate was used as the anticoagulant for the blood sample collections to minimize further hydrolysis of AS to DHA due to plasma esterases [28]. The blood sample was centrifuged at 1180×*g* for 5 min and plasma was separated. For patients with vivax malaria, heparinized venous blood (0.25 mL) was collected from the participants before CQ administration (day 0) and at day 28 after commencement of the CQ treatment regimen. Both blood and plasma samples were stored in liquid nitrogen at the field site. The samples were transferred to ADFMIDI on dry-ice and stored at − 80 °C until drug analysis.

### Anti-malarial drug analysis

Plasma concentrations of AS and LUM and their active metabolites (DHA and LUMm) concentrations and blood CQ and CQm concentrations were measured by liquid chromatography–mass spectrometry (LC/MS/MS) at ADFMIDI for the majority of patients. The limit of quantification for AS, DHA, LUM, and LUMm, CQ and CQm were 1.19 ng/mL, 1.96 ng/mL, 2.0 ng/mL, 1.0 ng/mL, 0.5 ng/mL and 0.5 ng/mL, respectively. For quality assurance, ADFMIDI participates in the WWARN proficiency testing/QC program for the measurement of plasma concentrations of the six analytes [29]. The chromatographic conditions, LC/MS/MS instrumentation settings, extraction method and the precision of the assays are outlined in Additional file 2.

### Statistical analysis

All data was analysed using SigmaStat (version 3.0 Jandel Scientific, CA, USA). Descriptive statistics were used to summarize baseline values and demographic data. Demographic and efficacy data were assessed by means of a per-protocol analysis, with recipients of rescue treatment counted as failures and new infections as cured. In vitro drug susceptibility and drug concentrations data are presented as median values (interquartile range; IQR). The parasite clearance half-life (PC_50_) was assessed with ≥ 3 parasite counts using the parasite clearance estimator [30]. For determining the confidence interval for small sample estimates the Adjusted Wald Method using an online web-based implementation developed by Lewis and Sauro [31] was applied. The Adjusted Wald Method was originally developed by Agresti and CoulI [32] and is recommended for small sample sizes (< 150) and binary data (pass/fail). The best estimate was determined using the Laplace estimation [33]. Statistical significance was defined as a *p *< 0.05.

## Results

### Patients with falciparum malaria treated with AS + DHA–PPQ or AM–LUM

Twenty-seven subjects were enrolled into the study and randomized to receive either AS + DHA–PPQ (n = 13) or AM–LUM (n = 14). At enrolment, the two treatment groups had similar demographic and clinical characteristics (Table 1). Of the patients recruited, 23% (3/13) on AS + DHA–PPQ and 29% (4/14) on AM–LUM were children (< 15 years of age). When combining parasitaemia in children and adults the geometrical mean parasitaemia was comparable between the two treatment groups (17,443 parasites/µL for AS + DHA–PPQ and 16,030 parasites/µL for AM–LUM). At the time of admission, 54% (7/13) of patients on AS + DHA–PPQ and 43% (6/14) on AM–LUM were febrile.

**Table 1:** Demographic and clinical characteristics of study participants before treatment with AS + DHA–PPQ and AM–LUM

Study group	AS + DHA–PPQ		AM–LUM	
	Children	Adults	Children	Adults
No. patients	3	10	4	10
Mean (SD) age (years)	11.7 ± 3.2	28.5 ± 12.5	8.3 ± 2.2	35.6 ± 10.5
Mean (SD) weight (kg)	29.8 ± 10.4	44.4 ± 5.8	15.4 ± 5.0	48.6 ± 4.9
Mean (SD) temp (°C)	38.0 ± 2.1	38.2 ± 0.6	38.4 ± 1.2	37.7 ± 0.6
No. patients temp ≥ 38 °C	1 (33%)	6 (60%)	3 (75%)	3 (30%)
Geometric mean parasites/µL (range)	16,200 (5227–84,459)	17,835 (4067–118,248)	16,884 (2014–88,144)	15,700 (2078–87,123)

Overall, one patient in each treatment group did not fulfil the per-protocol requirements. One adult male on AS + DHA–PPQ could not be located on days 35 and 42, and was thus lost to follow-up. PCR analysis of filter paper blood spots collected from participants before AS + DHA–PPQ and AM–LUM treatment revealed that one male adult had a mixed *P. falciparum*/*P. vivax* infection on day 0 of AM–LUM treatment with a recurrence of *P. vivax* malaria on day 42 of follow-up.

Both treatment regimens rapidly reduced parasitaemia, with only one patient on AM–LUM requiring 60 h to be blood film negative. AS and AM–LUM rapidly reduced the parasite load with a median 97.4% (range: 75.9–100%) and 71.3% (range: 6.7–94.1%) reduction in parasitaemia after 12 h following the first dose, respectively. The proportion of patient positive by blood film microscopy on days 1, 2, and 3 were: 69.2% (9/13) on day 1 and 0% for days 2 and 3 for the AS + DHA–PPQ group and 78.6% (9/14) for day 1, 7.1% (1/14) on day 2 and 0% on day 3 for the AM–LUM group. No significant difference was seen between the median parasite clearance time (*p* = 0.23; Mann–Whitney U test) for patients treated with AS (36 h; IQR 24–36 h) or AM–LUM (36 h; IQR 33–48 h). The PC_50_ was significantly shorter (*p* = 0.002 Mann–Whitney U test) in the AS + DHA–PPQ group (median 2.5 h; range: 0.4–5.4 h) compared with the AM–LUM group (median 5.4 h, range: 2.0–23.9 h).

All patients were afebrile by 36 h after commencing treatment with either AS or AM–LUM. After a 42-day follow-up period, no recurrence of falciparum malaria infection was observed in the 12 patients treated with AS + DHA–PPQ and in the 14 patients treated with AM–LUM indicating that both treatment regimens were highly effective. When applying a per-protocol analysis, the cure rate was 100% for both treatment regimens, with a 95% confidence interval of 88.4–100% and a best estimate of 96.3%.

The two treatment regimens were well tolerated, with no serious adverse events reported. Rigors/chills, sweating, headache, nausea, loss of appetite, fatigue and myalgia were the most common adverse events reported before treatment (Additional file 3: Table S1). With the exception of headache, loss of appetite and fatigue, the other symptoms declined markedly 24 h after starting treatment with most patients free of adverse events within 2–3 days after commencing treatment with either AS + DHA–PPQ or AM–LUM. Overall, the adverse events reported were generally mild in intensity before and after drug treatment.

### Molecular assays for artemisinin and piperaquine resistance

Sequencing of the 850 bp of *P. falciparum kelch*-13 gene, which covers the region corresponding to previously identified mutations from amino acid 427 to 687 were performed on the patient’s admission parasites. No polymorphisms were revealed (such as C580Y, R539T, Y493H and I543T) in the *pfkelch*-13 gene in 26 of the 27 patients. Also, no *exo*-*E415G* gene polymorphism on chromosome 13 or amplification in *plasmepsins 2* and *3* was observed in the *P. falciparum* parasites obtained from the 27 patients.

### In vitro drug susceptibility of clinical field isolates

**Fig. 1:**
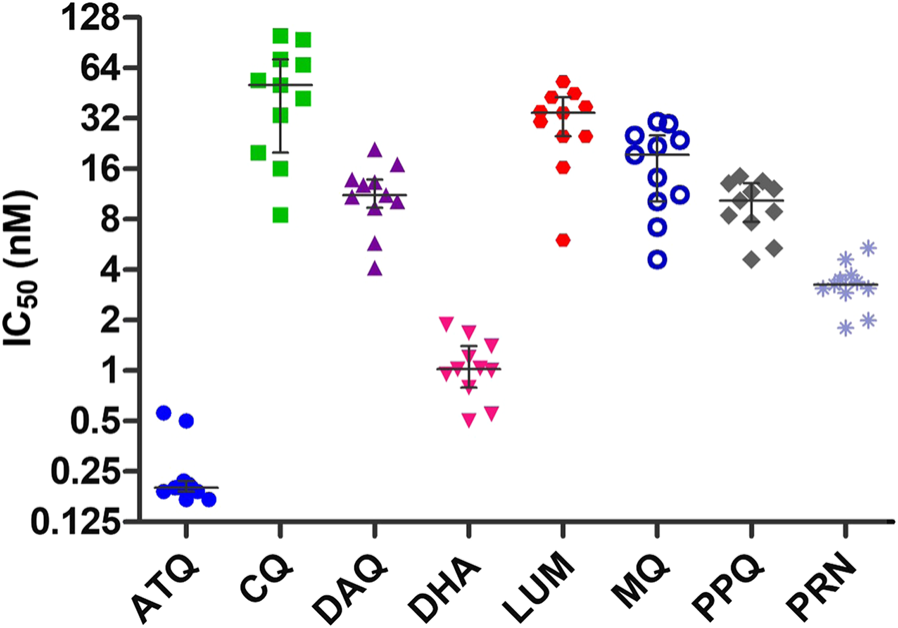
Median [interquartile range] IC_50_ (nM) values of standard anti-malarial drugs against *P. falciparum* isolates collected from 11 patients from Phuoc Chien Commune prior to treatment with either AS + DHA–PPQ (n = 5) or AM–LUM (n = 6)

In vitro drug susceptibility testing was carried out on *P. falciparum* isolates collected from 11 adults. All isolates were successful cultured at ADFMIDI, with median IC_50_ (nM) values derived from at least two independent experiments (Fig. 1). The median (range) IC_50_ values for the 11 *P. falciparum* isolates were as follows: 0.20 nM (0.17 to 0.56 nM) for ATQ, 51 nM (8.5 to 99.5 nM) for CQ, 11.2 nM (4.1 to 21 nM) for DAQ: 1.0 nM (0.5 to 1.9 nM) for DHA; 34.5 nM (6 to 53 nM) for LUM; 19.4 nM (4.6 to 31 nM) for MQ; 10.4 nM (4.6 to 14.5 nM) for PPQ and 3.3 nM (1.8 to 5.4 nM) for PRN. For comparison purposes, the in vitro drug susceptibility profile of the laboratory control *P. falciparum* drug-sensitive D6 and multidrug-resistant MRA1240 lines and the 11 participant’s field isolates are shown in Additional file 4: Table S1. The starting parasitaemia and parasite clearance times of the 11 participant’s that provided the field isolates of *P. falciparum* are outlined in Additional file 4: Table S2.

The in vitro findings showed that in the RSA the survival rates ranged between 0.7 and 1.5% (< 10.88% median cut-off, IQR: 4.75–13.91) for all five *P. falciparum* isolates that were collected from patients treated with AS–DHA + PPQ, greater than those for MRA1239^S^ (0.16%), but markedly less than that for MRA1240^R^ (7.3%). In the PSA, the same five isolates and both MRA lines were sensitive to PPQ, with survival rates ranging from 1.8 to 4.0% (< 10% cut-off). The results for the RSA and PSA are shown in Additional file 4: Tables S3 and S4).

### Patient drug concentrations following AS + DHA–PPQ or AM–LUM treatment

Patients plasma concentrations of the parent drugs (AS and LUM) and their active metabolites exhibited wide interpatient variability. The median (IQR) plasma concentration in 12 of 13 patients was 38.9 ng/mL (26.7–60.6 ng/mL) for AS and 777.4 ng/mL (225.2–1196.0 ng/mL) for DHA at 1 h after the last dose of AS on day 4. For determining exposure to AM–LUM treatment a day 7 blood sample was collected from 14 patients. The median (IQR) plasma concentration was 613.8 ng/mL (474.7–803.7 ng/mL) for LUM and 14.1 ng/mL (12.4–16.1 ng/mL) for LUMm. The mean (SD) day 7 metabolic ratio of LUMm/LUM was 2.4 ± 0.9%.

### Patients with vivax malaria treated with CQ

Of the 16 patients who participated in the study seven where children and nine were adults. The demographic and clinical characteristics of the patients treated with CQ at admission are shown in Table 2. The geometric mean parasitaemia for children was 1.25-fold lower than that seen for adults (3638 parasites/µL versus 4551 parasites/µL). The median parasite clearance time was 36 h [IQR 30–36 h] when combining responses for both children and adults after starting CQ treatment. No significant difference was seen between the median parasite clearance time (*p *= 0.10 Mann–Whitney U test) for children (36 h; IQR 24–36 h) and adults (36 h; IQR 36–48 h). CQ rapidly reduced the parasite load with a median 88.8% (range: 63.8–97.7%) reduction in parasitaemia 12 h after the first dose and by day 2 no parasites were detected in all participants blood films. The median PC_50_ in the CQ group was estimated at 4.7 h; with a range of 1.8–21.8 h. All patients were afebrile by 36 h after starting CQ treatment. The CQ treatment regimen was well tolerated, with no serious adverse events reported. The most common adverse events reported before treatment were rigors/chills, sweating, headache, nausea, loss of appetite, fatigue and myalgia (Additional file 5: Table S1). Many of these adverse events declined markedly 24 h after starting treatment, and by day 3 most patients were asymptomatic or presented with only mild adverse events.

**Table 2:** Demographic and clinical characteristics of study participants before treatment with CQ

Study group	Children	Adults
No. patients	7	9
Mean (SD) age (years)	8.0 ± 2.2	42.6 ± 14.5
Mean (SD) weight (kg)	19.5 ± 4.4	44.7 ± 7.3
Mean (SD) temp (°C)	37.6 ± 1.4	37.6 ± 0.7
No. patients temp ≥ 38 °C	3 (43%)	4 (44%)
Geometric mean parasites/µL, (range)	3638 (431–20,433)	4551 (530–14,387)

Sixteen patients enrolled in this study completed the CQ treatment with all but one completing the 28 days of follow-up and one patient diagnosed with *P. vivax* malaria on day 28 by microscopy blood film. Based on subsequent PCR analysis, all 16 patients at admission (day 0) had a mono-infection of *P. vivax* malaria. One patient was confirmed to have *P. vivax* at day 28 and another patient determined co-infected with *P. falciparum*/*P. vivax* on day 28. Thus, when applying per-protocol analysis the cure rate was 86.7% (13/15) for CQ, with a 95% confidence interval of 60.9–97.5% and a best estimate of 82.5%.

### Patient drug concentrations following CQ treatment

Of the 16 participants who enrolled in the study, 15 patients provided a blood sample before CQ administration (day 0) and of these 1/15 (6%) had measurable CQ and CQm concentrations of 2.1 ng/mL and 1.2 ng/mL, respectively. By day 28 the median (IQR) was 16.7 ng/mL (9.7–31.2 ng/mL, n = 15) for CQ and 25.8 ng/mL (15.4–31.6 ng/mL, n = 15) for CQm. The patient that had a recurrence of *P. vivax* infection on day 28 had CQ and CQm concentrations of 5.2 ng/mL and 7.5 ng/mL, respectively. Corresponding concentrations for the patient that was diagnosed with a mixed infection of *P. falciparum* and *P. vivax* on day 28 were 34.3 ng/mL and 31.7 ng/mL.

## Discussion

Reports of DHA–PPQ treatment failures in Binh Phuoc Province in south Vietnam and reduced artemisinin susceptibility in five other provinces in Vietnam, including Ninh Thuan Province, in treating uncomplicated falciparum malaria is of immense concern as other treatment options are limited. Until new drugs with different mechanisms of action to ACT are developed there is a need to evaluate other artemisinin-based combinations and to continue monitoring for reduced artemisinin susceptibility and DHA–PPQ failures nationwide and in the region.

The WHO’s definition of artemisinin resistance is a delay in parasite clearance following treatment with either AS monotherapy or with an artemisinin-based combination, which represents partial/relative resistance [6]. The delay in parasite clearance with ACT could lead to an increase in the risk of de novo resistance to the partner drug. Further evidence for artemisinin resistance are specific *pfkelch13* mutations that are associated with delayed parasite clearance in vitro and in vivo [26, 27, 34, 35]. The WHO has defined suspected endemic artemisinin resistance as ≥ 10% of patients with persistent parasitaemia by microscopy at 72 h after the commencement of treatment [6].

In this study, both artemisinin and ACT resistance for the treatment of falciparum malaria at Phuoc Chien Commune in southern Vietnam was assessed. To evaluate artemisinin resistance, patients were treated with AS monotherapy alone for 4 days before receiving a standard 3-day course of DHA–PPQ. The reason to evaluate AS alone prior to administering DHA–PPQ was to determine the effectiveness of AS in clearing falciparum parasites without a partner drug. Four days of AS treatment was considered sufficient to assess the susceptibility of AS alone as it covers two blood stage cycles of drug exposure, which historically has been sufficient to clear sensitive parasites, even with a lower AS dose regimen (4 mg/kg on day 0, 2 mg/kg on day 1–6) [35].

Of the 13 patients that received the AS + DHA–PPQ regimen all rapidly cleared their infections with a median parasite clearance time of 36 h. Only one patient had parasites present by microscopy at 36 h but was assessed as no parasites seen by 48 h after commencing AS treatment. Similar parasite clearance was also seen in the patients administered AM–LUM. These clinical findings suggest a lack of reduced susceptibility of AS and AM–LUM in the treated patients. Although these results are encouraging for the residents of Phuoc Chien Commune this was not the case in nearby communes (i.e., within 60 km of Phuoc Chien Commune). In the same province in 2015 the number of *P. falciparum*-infected patients with parasitaemia on day 3 after starting DHA–PPQ has been reported to be 8% for Phuoc Thang Commune (Bac Ai District) and 11% for Manoi Commune (Ninh Son District). These studies were carried out by the Oxford University Clinical Research Unit/Institute of Malariology, Parasitology and Entomology (IMPE) for Phuoc Thang Commune (Principal investigator, Ngo Viet Thanh, unpublished data) and WHO/IMPE for Manoi Commune (Principal investigator, Huynh Hong Quang, unpublished data).

Mutations in the *pfkelch*-*13* and *exo*-*E415G* genes are molecular markers of *P. falciparum* parasite resistance to artemisinins and piperaquine, respectively. For the *pfkelch*-*13* gene, polymorphisms associated with amino acid changes C580Y, R539T, Y493H and I543T in the eastern Greater Mekong Subregion (GMS–Cambodia, Lao and Vietnam) and F446L, N458Y, P574L and R561H in the western GMS (China, Myanmar and Thailand) have been linked to artemisinin resistance [6]. None of these changes in *pfkelch*-13 gene were seen in the participants *P. falciparum* parasites on either treatment regimens. Also, no polymorphism in *exo*-*E415G* and single copies of *plasmepsins 2* and *3* were observed in the participants’ *P. falciparum* parasites.

In vitro drug susceptibility testing has routinely been used to monitor for anti-malarial drug resistance. Using the standard hypoxanthine incorporation assay the threshold IC_50_ values for *P. falciparum* in vitro resistance or decreased susceptibility to CQ, DAQ and MQ are > 100 nM, > 60 nM and > 30 nM, respectively [36], > 110 nM for LUM [37, 38] and > 28 nM for ATQ [39]. Cut-off IC_50_ values for reduced susceptibility to PRN and PPQ are > 60 nM and > 135 nM, respectively [40]. Based on these cut-off values the *P. falciparum* isolates collected from the 11 participants revealed that they were susceptible to CQ, DAQ, PRN, PPQ, MQ, LUM and ATQ. These in vitro findings further support the clinical data, particularly with the participants’ *P. falciparum* isolates being susceptible to the long acting partner drugs of LUM and PPQ. However, with the hypoxanthine incorporation assay there is a substantial overlap in the distribution of IC_50s_ for DHA between rapidly and slowly cleared parasites, with the later implicated in DHA resistance [26]. The RSA and PSA are the preferred in vitro assays for predicting clinical failures for DHA–PPQ [26, 27, 41]. For the five *P. falciparum* isolates collected from patients before treatment with AS + DHA–PPQ, all were susceptible to DHA and PPQ.

In the evaluation of artemisinin resistance a potential confounding factor that needs to be addressed is insufficient blood drug concentrations or reduced patient drug exposure. In the present study, a 1 h post last dose of AS was selected as this is close to the maximum plasma concentration of DHA after AS dosing and the levels obtained in the participants were comparable with published values [42–45].  Plasma concentration of LUM on day 7 has been found to be a good surrogate of drug exposure (i.e., area under the plasma drug concentration curve) and has been identified as a major determinant of therapeutic response to AM–LUM [46]. The plasma LUM and LUMm concentrations were similar to published data [46–49] and 79% (11/14) of patients had LUM concentrations above the day 7 threshold concentration of 280 ng/mL, which is associated with an increased risk of recrudescence [46]. The plasma concentrations of AS and LUM and their major metabolites provide evidence of good drug exposure after both AS and AM–LUM treatments.

Unlike falciparum malaria whereby PCR methods are used to differentiate recrudescence and reinfection, distinguishing recrudescence from reinfection or relapse is still not possible for vivax malaria [9]. The first relapse wave of tropical Asian strains of *P. vivax* malaria tends to occur on or before day 28 following a standard 3-day course of CQ (total 25 mg/kg) [9]. The 3–4 weeks relapse period appears to be the case for *P. vivax* malaria in Vietnam with other studies [10, 11] reporting recurrent parasitaemia within 28 days after starting CQ treatment. A predictor of a highly CQ-resistant strain of *P. vivax* is persistent or recurrent parasitaemia within 14 days of starting a standard treatment course of CQ [50]. Additionally, a key in demonstrating CQ-resistant *P. vivax* malaria is the presence of blood CQ concentrations at the time of an unremitting or recurrent parasitaemia in excess of the minimally effective concentration established for sensitive *P. vivax* strains. The putative minimal blood composite CQ + CQm concentration for CQ-sensitive *P. vixax* malaria is 100 ng/mL at day 28 after the commencement of a standard 3-day course of CQ [9, 50, 51].

The first report of CQ-resistance in Vietnam for the treatment of vivax malaria was that by Phan et al. [10] with a failure rate of 16%, but in that study blood CQ and CQm concentrations were not measured to eliminate the confounding factor of insufficient drug exposure in the participants. In this study, the minimum composite value was not achieved in any participant, with a median [IQR] CQ + CQm of 44.6 ng/mL [25.3–67.4 ng/mL] at day 28 for the 14 participants. Of note, low and comparable blood CQ + CQm concentrations was also measured in Vietnamese patients treated with CQ for *P. vivax* malaria in Binh Phuoc Province, with a median CQ + CQm concentrations of 46.1 ng/mL (n = 51) at day 28 [12]. By contrast, a study in Quang Nam Province revealed 3 of 8 Vietnamese patients who had recurrent vivax malaria after CQ treatment with interpretable results had blood CQ concentrations above 100 ng/mL [11].

The low CQ and CQm blood concentrations measured in this study and that of Thuan et al. [12] is of concern as it could compromise the curative efficacy of CQ in Vietnam. For both Vietnamese studies compliance was absolute with direct observed therapy of CQ tablets (dosing of 25 mg/kg) provided by the Vietnam National Malaria Control Programme. Additionally, using LC/MS/MS we determined that the mean CQ content of the tablets manufactured by Mekophar Chemical Pharmaceutical Joint Stock Company was 93.6 ± 7.5% (n = 16 tablets) of the nominal amount of 150 mg of CQ base. Analogous studies in Indonesia [52] and Tanzania [53, 54] in patients with vivax and falciparum malaria, respectively, have also reported blood CQ + CQm concentrations at day 28 following the commencement of the standard CQ therapy with composite values less than 100 ng/mL. Variations in the pharmacokinetics of CQ in different racial/ethnic groups and unique dietary practices may explain why some ethnic groups may have lower CQ + CQm concentrations at day 28 of follow-up [52].

In the present study, two patients had a recurrence of *P. vivax* infection on day 28 with CQ + CQm concentrations of 12.7 ng/mL and 66.0 ng/mL. As their concentrations were well below the minimal CQ + CQm concentrations of 100 ng/mL these *P. vivax* infections cannot be judged as CQ-resistant, particularly the patient that had the lowest concentration (indicating poor drug absorption). However, with the other 11 patients who did not experience a recurrence of parasitaemia at Phuoc Chien Commune it appears that the *P. vivax* parasites at the field site are still CQ-sensitive, despite having low blood CQ exposure. Because all patients treated with CQ did not experience a recurrence of vivax malaria within 14 days of starting CQ treatment, they were likely not infected with a highly CQ-resistant *P. vivax* strain.

A limitation of the two therapeutic efficacy studies was the low number of participants which was reflected in the overall marked decline in malaria cases in Vietnam from 9331 in 2015 and to 4161 in 2016 [1]. It is suspected that the decline in the country’s prevalence of malaria including at Phuoc Chien Commune were due to several factors, such as improved social-economic conditions, with less ethnic minority populations working in forested regions of the country where malaria transmission is present, government restrictions to people working in forested areas and movement of people across border regions of the country, and greater access and adoption of effective intervention methods, such as insecticide-impregnated bed nets and improved case management with ACT [55]. Nonetheless, the clinical and laboratory findings in the present study provide an insight into drug susceptibility at the field site in Ninh Thuan Province.

## Conclusions

Despite the low number of participants in the two efficacy studies the clinical findings following AS + DHA–PPQ or AM–LUM treatment of patients with *P. falciparum* malaria at the field site in Ninh Thuan Province substantiated by molecular analysis of drug resistance, in vitro drug susceptibility testing of field isolates and drug analysis providing evidence of adequate drug exposure suggest that the falciparum parasites were susceptible to AS and ACT. Vivax malaria also appears to be susceptible to CQ at the field site but the low patient blood CQ concentrations revealed in this study needs to be further evaluated to provide definitive data on the efficacy of CQ treatment.

## Additional files

**Additional file 1: Figure S1.** Map of Vietnam showing location of Ninh Thuan Province (A) and location of Phuoc Chien Commune in Thuan Bac District, Ninh Thuan Province (B).

**Additional file 2.** Summary of drug analysis of artesunate, dihydroartemisinin, lumefantrine, desbutyl-lumefantrine, chloroquine and desethyl-chloroquine.

**Additional file 3: Table S1.** Adverse events before and after treatment of Vietnamese patients with AS + DHA–PPQ or AM–LUM for uncomplicated falciparum malaria.

**Additional file 4: Table S1.** In vitro drug susceptibility data (IC_50_, nM) of the laboratory control *Plasmodium falciparum* D6 and MRA1240 lines and the 11 field isolates of *Plasmodium falciparum* collected from study participants. **Table S2.** Admission parasitaemia and parasite clearance time of the 11 adult patients who provided a blood sample for in vitro drug susceptibility testing (see Table S1 for IC_50_ values). **Table S3.** Determination of the proportion of viable parasites (% survival) in DHA-treated compared to untreated cultures by microscopy. **Table S4.** Determination of the proportion of viable parasites (% survival) in PPQ-treated compared to untreated cultures by microscopy.

**Additional file 5: Table S1.** Adverse events before and after treatment of Vietnamese patients with CQ for uncomplicated vivax malaria.
