# Supplementary material for: Susceptibility of Plasmodium falciparum to artemisinins and Plasmodium vivax to chloroquine in Phuoc Chien Commune, Ninh Thuan Province, south-central Vietnam
Source: Malar J. 2019 Jan 17;18:10. doi: 10.1186/s12936-019-2640-2 (PMC6335800; doi:10.1186/s12936-019-2640-2)
Supplement: Supplementary file 1 — Additional file 1: Figure S1. Map of Vietnam showing location of Ninh Thuan Province (A) and location of Phuoc Chien Commune in Thuan Bac District, Ninh Thuan Province (B). [file 12936_2019_2640_MOESM1_ESM.docx]

**Additional file 1:**

**Fig S1.** Map of Vietnam showing location of Ninh Thuan Province (A) and location of Phuoc Chien Commune in Thuan Bac District, Ninh Thuan Province (B).

| 1. **Map of Vietnam showing Ninh Thuan Province** | 1. **Map of Thuan Bac District in**   **Ninh Thuan Province** |
| --- | --- |
| 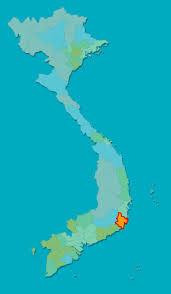 | 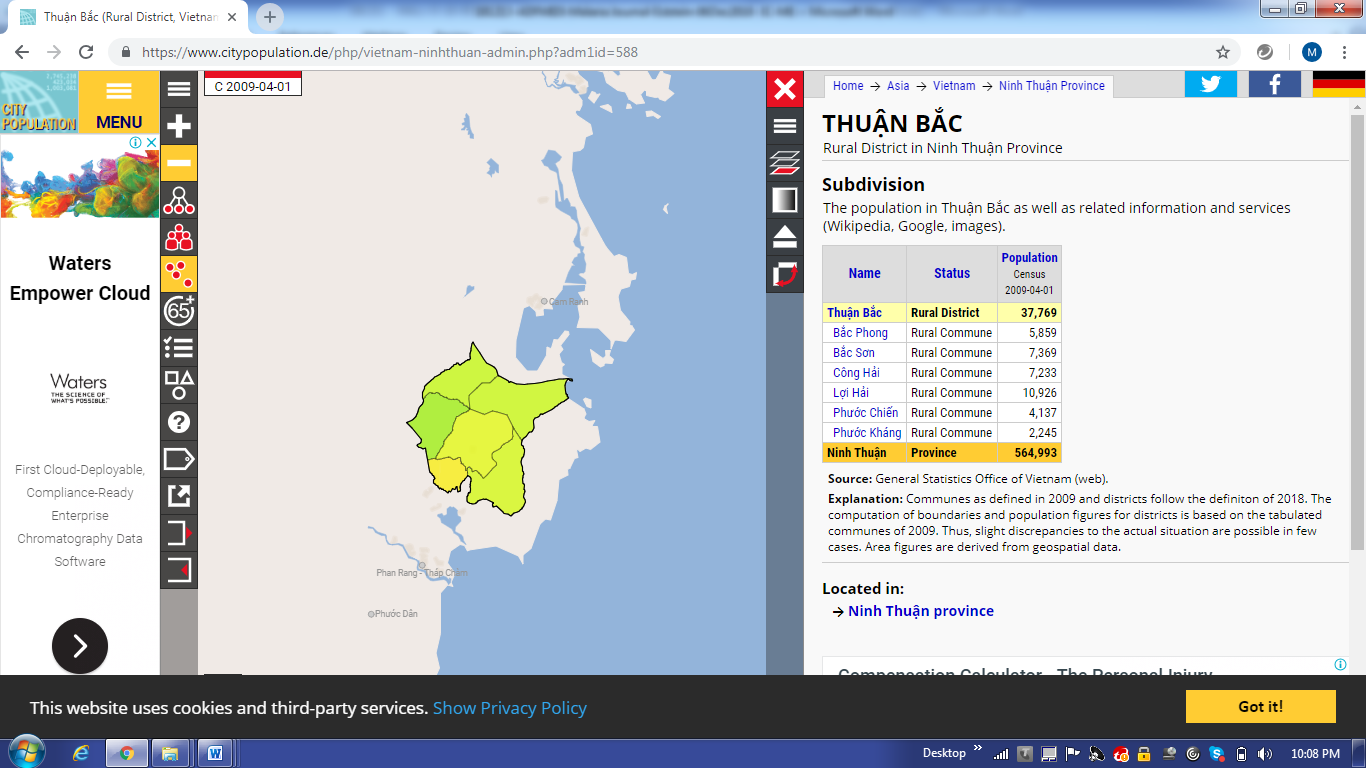  **Phuoc Chien Commune** |
