# Supplementary material for: Susceptibility of Plasmodium falciparum to artemisinins and Plasmodium vivax to chloroquine in Phuoc Chien Commune, Ninh Thuan Province, south-central Vietnam
Source: Malar J. 2019 Jan 17;18:10. doi: 10.1186/s12936-019-2640-2 (PMC6335800; doi:10.1186/s12936-019-2640-2)
Supplement: Supplementary file 2 — Additional file 2. Summary of drug analysis of artesunate, dihydroartemisinin, lumefantrine, desbutyl-lumefantrine, chloroquine and desethyl-chloroquine. [file 12936_2019_2640_MOESM2_ESM.docx]

**Additional file 2:**

**Summary of drug analysis of artesunate, dihydroartemisinin, lumefantrine, desbutyl-lumefantrine, chloroquine and desethyl-chloroquine**

Plasma concentrations of artesunate (AS), dihydroartemisinin (DHA), lumefantrine (LUM), and desbutyl-lumefantrine (LUMm) and blood concentrations of chloroquine (CQ) and desethyl-chloroquine (CQm) were measured on an AB Sciex 4000 QTRAP LC-MS/MS analyser (Applied Biosystems) preceded by a Prominence liquid chromatography system (Shimadzu), including a temperature-controlled autosampler. The mobile phases were subjected to electrospray ionisation in the positive ion mode and elution of analytes was confirmed by multiple-reaction monitoring (MRM). Protein precipitation using acetonitrile was applied for sample preparation and either SIL-AS, SIL-DHA, LUMa, and CQa were used as internal standards for their respective parent drug and metabolites.

In brief, the lower limit of quantification (LLOQ) of AS and DHA in plasma was 1.19 ng/mL and 1.96 ng/mL (n = 7), respectively, with an inaccuracy of ≤2.1%, using 50 µL of sample. The inter-assay precision of analysis (percent coefficient of variation [CV]) for AS and DHA across the concentration range of 1.19 ng/mL to 728 ng/mL (n=7) and 1.96 ng/mL to 2,500 ng/mL (n = 7-9), respectively, was ≤8.8%. The LLOQ of LUM and LUMm in plasma was 2.0 ng/mL and 1.0 ng/mL (n = 13), respectively, with an inaccuracy of ≤0.7%, using 50 µL of sample. The inter-assay CV for LUM and LUMm across the concentration range of 2 ng/mL to 3,000 ng/mL (n=9-13) and 1.0 ng/mL to 2,000 ng/mL (n = 9-13), respectively, was ≤6.0%. The LLOQ of CQ and CQm in blood was 0.5 ng/mL for both analytes (n = 8), with an inaccuracy of ≤1.9%, using 50 µL of sample. The inter-assay CV for CQ and CQm across the concentration ranges of 0.5 ng/mL to 1,000 ng/mL (n=7-9) was ≤9.2%.

- 1. **LC/MS/MS analysis of AS and DHA**
     1. **LC/MS/MS Conditions**

Sample extracts were analysed on an AB Sciex 4000 QTRAP LC-MS/MS analyser (Applied Biosystems) preceded by a Prominence liquid chromatography system (Shimadzu), including a temperature-controlled autosampler.

- - 1. **Chromatographic Conditions**

LC-Column: Hypersil Gold C18 (150 × 2.1 mm, 5 μm) column

Pre-Column: Hypersil Gold C18 (10 × 2.1 mm, 3 μm) guard cartridge (Thermo Electron Corporation, Madison, WI, USA)

Mobile Phase A: 5 mM NH_4_ Acetate, 0.5% Acetic Acid, 50% Acetonitrile

Mobile Phase B: Methanol:Acetonitrile (3:1)

Mobile Phase Flow: Gradient as follows:

| Time | Module | Events | Parameter |
| --- | --- | --- | --- |
| 0.30 | Pumps | Pump B Conc. | 0.0 |
| 0.80 | Pumps | Pump B Conc. | 100 |
| 2.20 | Pumps | Pump B Conc. | 100 |
| 2.40 | Pumps | Total Flow | 1 |
| 3.30 | Pumps | Total Flow | 1 |
| 3.50 | Pumps | Pump B Conc. | 0 |
| 3.50 | Pumps | Total Flow | 0.75 |
| 5.20 | Pumps | Pump B Conc. | 0.0 |
| 5.40 | Pumps | Total Flow | 0.5 |
| 5.50 | System Controller | Stop |  |

Column Oven: 40°C

Detector: ABSciex 4000 Qtrap LCMS Spectrophotometer

Injector Rinse Sol: 30% Acetonitrile

Auto Sample Chamber Temp: 4-8°C

Regression: ABSciex Multiquant 2.1 MP4 integration with quadratic regression analysis with a weighting of 1/x^2^

- - 1. **MS Conditions**

Source conditions

| Curtain gas | 25 |
| --- | --- |
| CAD gas | High |
| IS (V) | 5,500 |
| Temp (˚C) | 300 |
| GS1 | 50 |
| GS2 | 50 |
| IHE | On |

MS Transitions:

| Analyte | Q1  (Da) | Q3  (Da) | Time  (msec) | DP  (v) | EP  (v) | CE  (v) | CXP  (v) |
| --- | --- | --- | --- | --- | --- | --- | --- |
| AS | 402.1 | 267.1 | 100 | 46 | 10 | 15 | 22 |
| DHA | 302.2 | 163.2 | 100 | 41 | 10 | 23 | 14 |
| SIL-AS | 406.0 | 163.0 | 100 | 40 | 10 | 16 | 12 |
| SIL-DHA | 307.0 | 168.0 | 00 | 36 | 10 | 23 | 12 |

- - 1. **Testing of Drug-Free Plasma (DFP) for Ion Suppression**

Six drug-free citrate phosphate dextrose anticoagulated human plasma (DFPs) were tested for matrix effect (ion suppression) to identify drug-free plasma that showed no identifiable effect on drug quantification. This was done by infusing a low concentration of AS, DHA, SIL-AS and SIL-DHA while injecting mobile phase extracts from the six DFPs. None of the plasmas showed ion suppression and one of these, was used to prepare calibrators and quality control (QC) samples.

- - 1. **Preparation of Calibration and Internal Standards**
- AS: Sample # RMARS 20100526-11. MW 384.42 g/mol. WWARN Reference compound.
- DHA: Sample # RMDHA 20100510-20. MW 284.35 g/mol. WWARN Reference compound.
- Internal standards: SIL-AS (stable isotope labelled AS) and SIL-DHA (stable isotope labelled DHA) were both obtained from WWARN, Bangkok.
- A quantity of AS was weighed and the amount of base compound calculated, a volume of methanol was added to make a stock solution of 1 mg/mL.
- A quantity of DHA was weighed and the amount of base compound calculated, a volume of methanol was added to make a stock solution of 1 mg/mL.
- The AS and DHA stock solutions were further diluted in 50% methanol to prepare intermediate solutions as per tables below.
- Preparation of Intermediate Solutions of AS.

| IS | Final concentation  (μg/mL) | Volume WS  (μL) | Ethanol-water  (50-50, v/v) (μL) |
| --- | --- | --- | --- |
| ISB_A1B_ | 72.8 | 110 stock  1 mg/mL AS | 1,400 |
| ISB_A2B_ | 22.4 | 400 WSB_1B_ | 900 |
| ISB_A3B_ | 6.90 | 400 WSB_2B_ | 900 |
| ISB_A4B_ | 2.12 | 400 WSB_3B_ | 900 |
| ISB_A5B_ | 0.385 | 200 WSB_4B_ | 900 |
| ISB_A6B_ | 0.119 | 400 WSB_5B_ | 900 |

- Preparation of Intermediate Solutions of DHA.

| IS | Final concentation  (μg/mL) | Volume WS  (μL) | Ethanol-water  (50-50, v/v) (μL) |
| --- | --- | --- | --- |
| ISB_D1B_ | 250 | 400 stock  1 mg/mL DHA | 1,200 |
| ISB_D2B_ | 62.5 | 400 WSB_1B_ | 1,200 |
| ISB_D3B_ | 15.6 | 400 WSB_2B_ | 1,200 |
| ISB_D4B_ | 3.91 | 400 WSB_3B_ | 1,200 |
| ISB_D5B_ | 0.782 | 300 WSB_4B_ | 1,200 |
| ISB_D6B_ | 0.196 | 400 WSB_5B_ | 1,200 |

- A quantity of SIL-AS and SIL-DHA was weighed and the amounts of base compounds calculated. Volumes of methanol were added to make stock solutions of 1 mg/mL each.
- Appropriate volumes of each stock solution were combined to produce a mixed intermediate stock solution of 1 µg/mL SIL-AS and 5 µg/mL SIL-DHA in 50% methanol/water.
- This solution was further diluted just before use, 1/500 in acetonitrile, to produce a mixture of final concentrations of 2 ng/mL SIL-AS and 10 ng/mL SIL-DHA as the internal standard working solution.
- Calibration standards containing both AS and DHA were prepared by spiking DFP with differing volumes of the intermediate solutions and performing serial dilutions to produce working solutions according to table below. Each intermediate solution was mixed before use. The working solutions were stored in 2 mL cryo vials at -80°C until analysis.
- Preparation of Combined Working Solutions of AS and DHA.

| WS | Final AS concentation  (μg/mL) | Final DHA concentation  (μg/mL) | Volume ISB_A_  (μL) | Volume ISB_D_  (μL) |
| --- | --- | --- | --- | --- |
| WSB_C1B_ | 36.4 | 125 | 400 ISB_A1B_ | 400 ISB_D1B_ |
| WSB_C2B_ | 11.2 | 31.3 | 400 ISB_A2B_ | 400 ISB_D2B_ |
| WSB_C3B_ | 3.45 | 7.81 | 400 ISB_A3B_ | 400 ISB_D3B_ |
| WSB_C4B_ | 1.06 | 1.95 | 400 ISB_A4B_ | 400 ISB_D4B_ |
| WSB_C5B_ |  | 0.391 | 400 ISB_A5B_ | 400 ISB_D5B_ |
| WSB_C6B_ |  | 0.0978 | 400 ISB_A6B_ | 400 ISB_D6B_ |

- Preparation of QC Plasma Samples

Quality control (QC) samples containing AS and DHA were prepared in DFP on ice covering a low, mid and high range .The QC samples were analysed in duplicate with each analytical batch in an identical manner to the calibration and unknown plasma samples. A quantification run was considered invalid if more than two QC samples observed values deviated by > ±15% of their calculated value, or if two QC samples from the same group, low, mid or high, deviated by > ±15%.

- Preparation of Calibration Standards and QCs of AS and DHA.

| WS | Final AS concentation  (ng/mL) | Final DHA concentation  (ng/mL) | Volume WSB_C_  (μL) | Volume plasma  (μL) |
| --- | --- | --- | --- | --- |
| Std 1 | 0 | 0 | 0 | 5,000 |
| Std 2 | 1.19 | 1.96 | 100 WSB_C6B_ | 4,900 |
| Std 3 | 3.86 | 7.82 | 100 WSB_C5B_ | 4,900 |
| Std 4 | 21.2 | 39.1 | 100 WSB_C4B_ | 4,900 |
| Std 5 | 69.0 | 156 | 100 WSB_C3B_ | 4,900 |
| Std 6 | 224 | 625 | 100 WSB_C2B_ | 4,900 |
| Std 7 | 728 | 2,500 | 100 WSB_C1B_ | 4,900 |
| QC1 | 2.90 | 5.87 | 75 WSB_C5B_ | 4,925 |
| QC2 | 51.7 | 117 | 75 WSB_C3B_ | 4,925 |
| QC3 | 546 | 1,880 | 75 WSB_C1B_ | 4,925 |

- Volumes of combined working solutions above were pipetted into suitable plastic tubes. The tubes were mixed and kept on ice for 30 min. 400 μL was aliquoted into eppendorf tubes and frozen at -80°C.
- During the LC run replicates of each QC level were analysed at the beginning and end of the sequence.
  - 1. **Sample Preparation**
- Calibrators and QCs were prepared by spiking AS and DHA in DFP (50 µL) with acetonitrile containing internal standards (2 ng/mL SIL-AS and 10 ng/mL SIL-DHA in acetonitrile; 200 µL), or blank acetonitrile (200 µL) for the double blank. Calibrators and blanks were then treated as unknowns.
- Subject plasma samples were thawed in a room temperature circulating water bath and vortex-mixed prior to analysis.
- Subject or spiked plasma (50 µL) were added to pre-labelled 1.5 mL polypropylene microcentrifuge tubes. Internal standard solution (2 ng/mL SIL-AS and 10 ng/mL SIL-DHA in acetonitrile; 200 µL) was added and the tubes mixed by vortexing for 1 min at maximum vortex setting.
- The tubes were then centrifuged (20,817 x *g*, 5 min at 4°C).
- The organic layer (100 µL) was subsequently transferred to a 96 well polypropylene microtitre plate containing 100 µL of 0.1% formic acid, and the assay plate sealed with a silicone mat.
- 10 µL was injected on column.
  - 1. **Quantification**

Chromatograms were integrated and processed using Applied Biosystems software (Analyst V1.4.2). Concentrations of DHA and ARS in subject plasma samples and quality controls were calculated from regression curves of peak area ratios (DHA:SIL-DHA and ARS:SIL-ARS) versus analyte concentration using Multiquant 2.1 software. A weighting of (1/X^2^) was used to generate a quadratic regression curve.

- - 1. **QA/QC**

Paired samples of each QC plasma sample were run with each batch.

- 1. **Chromatograms**
     1. **AS and DHA**

Typical chromatograms of AS, DHA and the internal standard SIL-AS and SIL-DHA in plasma are shown in Figures S1 to S6. The retention times for AS, DHA, SIL-AS and SIL-DHA were approximately at 1.49, 1.25, 1.49 and 1.25 min, respectively.

Figure S1. Drug-free plasma (DFP) sample for AS and DHA analysis

| 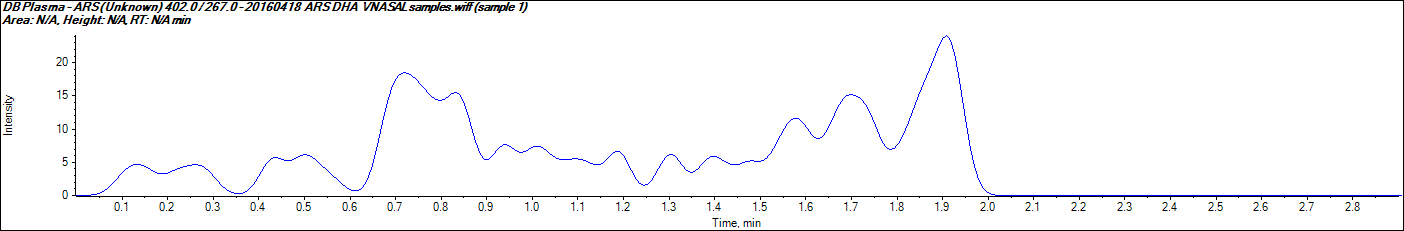 | 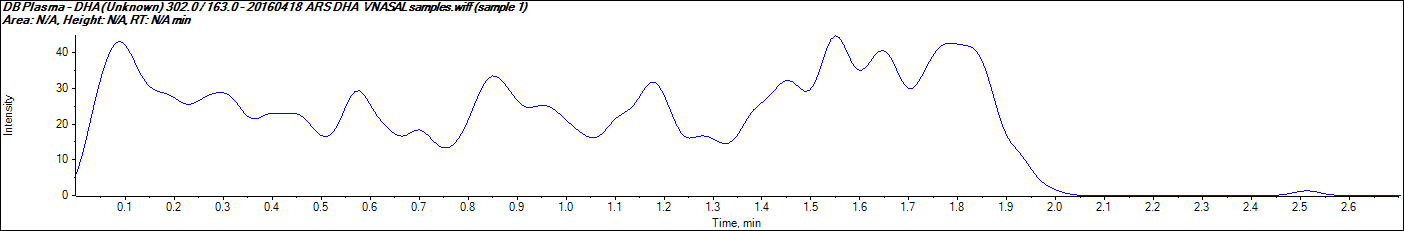 |
| --- | --- |

| Figure S2. Low-range QC plasma nominal concentrations of AS (2.9 ng/mL) and DHA (5.87 ng/mL)   \| 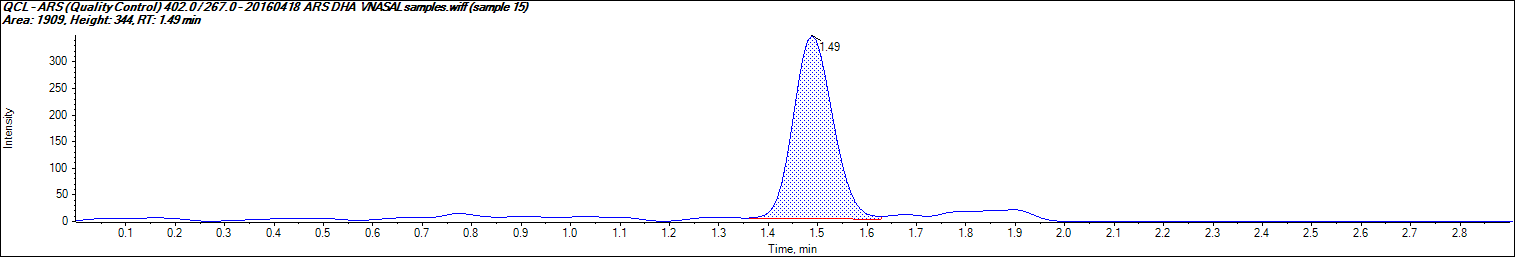 \| 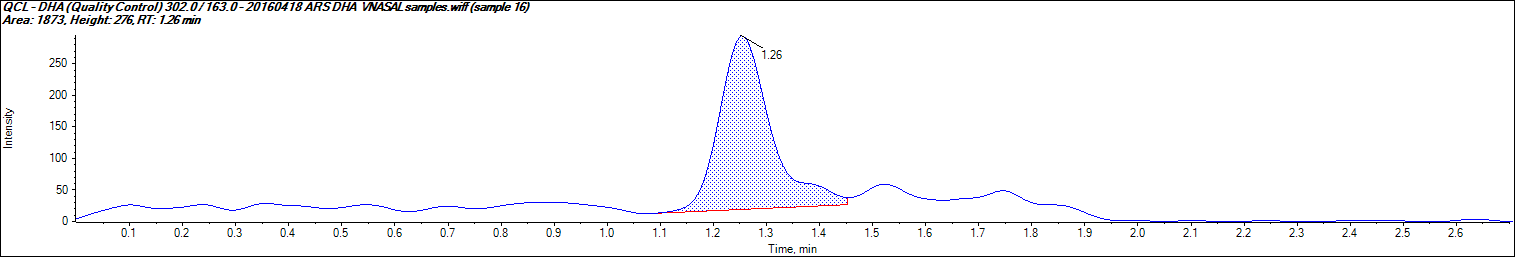 \| \| --- \| --- \| |
| --- | --- | --- |
| Figure S3. Spiked DFP containing nominal concentrations of AS (1.19 ng/mL) and DHA (1.96 ng/mL)   \| 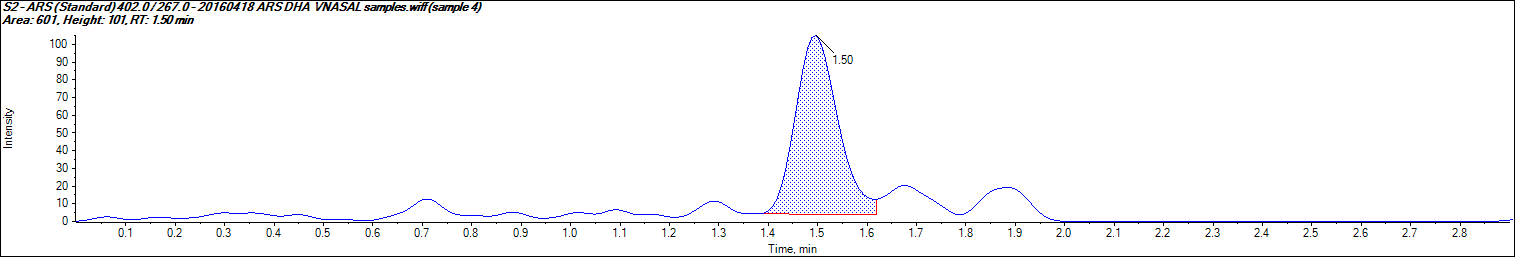 \| 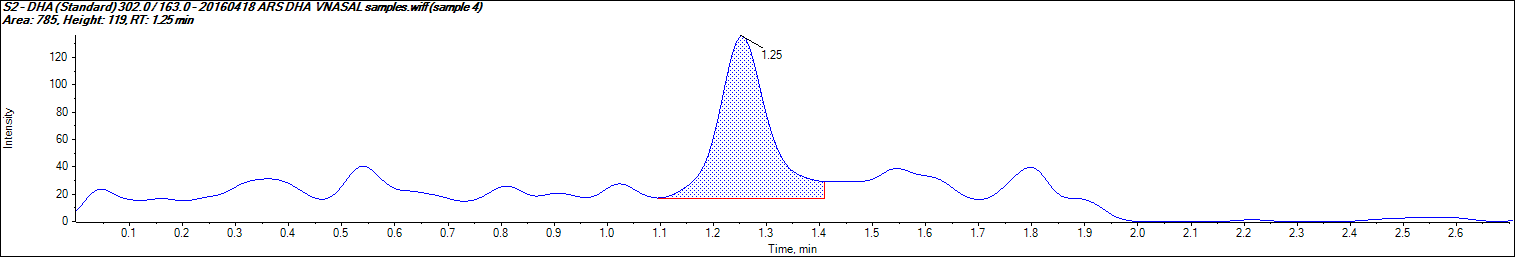 \| \| --- \| --- \| |

Figure S4. Spiked DFP containing nominal concentrations of AS (728 ng/mL) and DHA (2,500 ng/mL)

| 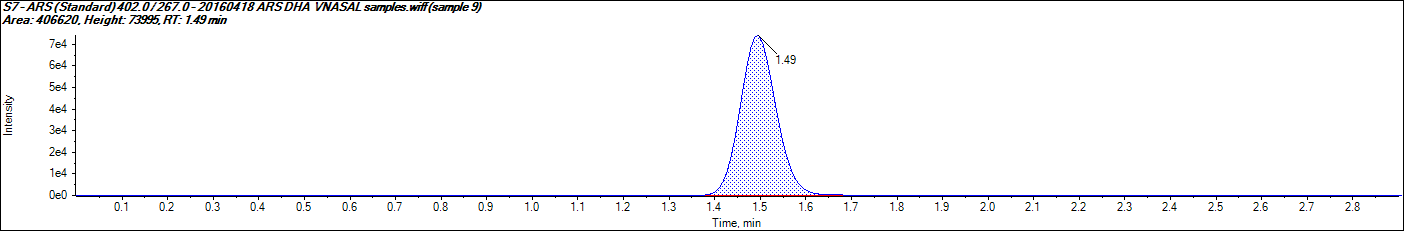 | 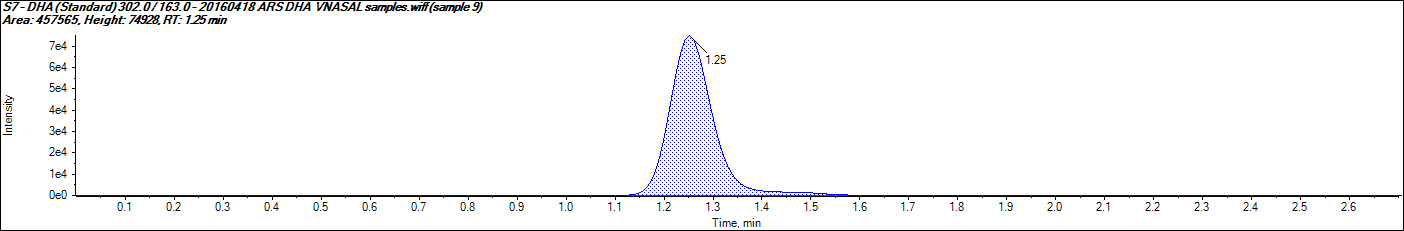 |
| --- | --- |

Figure S5. Plasma sample from patient VAS02A (AS 36.49 ng/mL; DHA 1547.35 ng/mL)

| **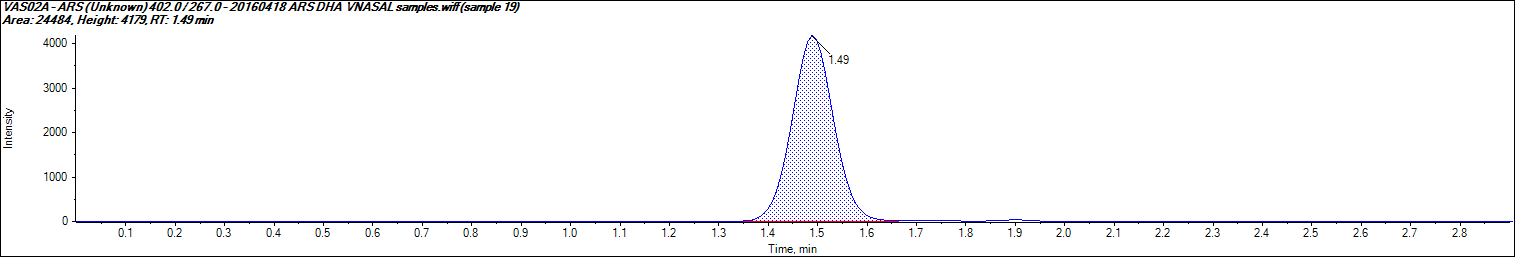** | **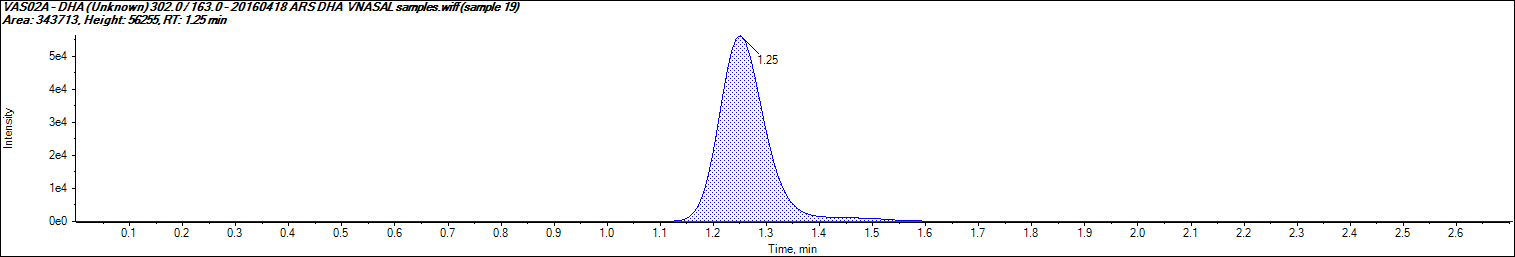** |
| --- | --- |

Figure S6. SIL-DHA and SIL-AS in extracted plasma sample for patient VAS02

| 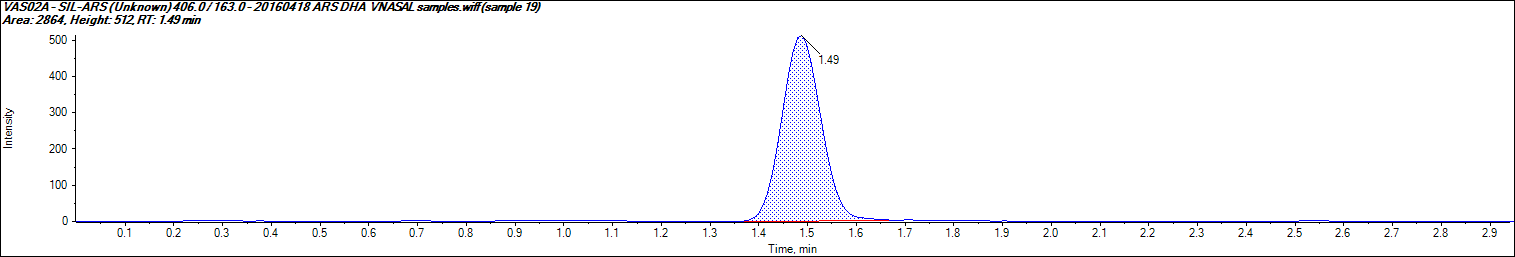 | 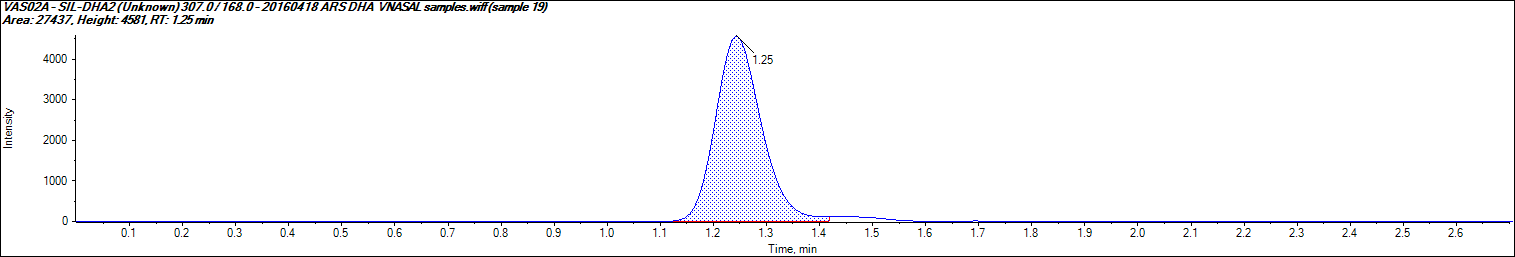 |
| --- | --- |

- 1. **Method Validation Plasma Samples**
     1. **AS and DHA**

1. **Accuracy and Precision**

Calibration standards of plasma AS and DHA concentrations from 1.19 to 728 and 1.96 to 2,500 ng/mL, respectively, were analysed on the different days (Table S1). Good linearity was obtained over this range, with good precision and accuracy.

Table S1. Inter-day Accuracy and Precision of AS and DHA assay.

| Nominal Conc. (ng/mL) | Mean (ng/mL) | SD (ng/mL) | CV  (%) | n | Mean  Accuracy (%) |
| --- | --- | --- | --- | --- | --- |
| AS | | | | | |
| 1.19 | 1.22 | 0.03 | 2.5 | 7 | 102.1 |
| 3.86 | 3.85 | 0.24 | 6.3 | 7 | 99.8 |
| 21.2 | 21.26 | 1.33 | 6.3 | 7 | 100.3 |
| 69.0 | 66.65 | 2.30 | 3.5 | 7 | 96.6 |
| 224 | 226.93 | 9.31 | 4.1 | 7 | 101.3 |
| 728 | 727.15 | 9.33 | 1.3 | 7 | 99.9 |
|  | | | | | |
| DHA | | | | | |
| 1.96 | 1.97 | 0.12 | 6.3 | 7 | 100.6 |
| 7.82 | 8.06 | 0.71 | 8.8 | 7 | 103.0 |
| 39.1 | 39.31 | 2.81 | 7.1 | 7 | 100.6 |
| 156 | 152.58 | 4.28 | 2.8 | 7 | 97.8 |
| 625 | 606.19 | 42.66 | 7.0 | 7 | 97.0 |
| 2,500 | 2,522.32 | 43.54 | 1.7 | 9 | 100.9 |

- For AS the lower limit of quantification (LLOQ) using 50 µL of plasma was 1.19 ng/mL, with a coefficient of variation (CV) of 2.5% and an accuracy of 102.1%.
- For DHA the lower limit of quantification (LLOQ) using 50 µL of plasma was 1.96 ng/mL, with a CV of 6.3% and an accuracy of 100.6%.
- A quadratic regression equation *y = ax^2^ + bx+ c* where *x* is the amount of drug and *y* is the peak area ratio, was used to determine the concentrations of unknowns and QC samples. A typical regression equation of a calibration curve was:

AS: a = 2.71346x10^-6^, b = 0.23307, c = 0.04149, with r = 0.99850

DHA: a = -9.75983x10^-8^, b = 0.00823, c = 0.02799, with r = 0.99637

1. **QC Plasma Samples**

Paired QC samples for low, mid and high concentrations from were included with each analytical batch. Results of accuracy and precision of QC samples (mid-low-high concentrations) that fell within acceptance criteria are shown in Table S2.

Table S2. Accuracy and Precision of plasma AS and DHA QC samples

| Nominal Conc. (ng/mL) | Mean (ng/mL) | SD (ng/mL) | CV (%) | n | Mean Accuracy (%) |
| --- | --- | --- | --- | --- | --- |
| AS | | | | | |
| 2.9 | 3.00 | 0.21 | 6.9% | 9 | 103.5 |
| 51.7 | 51.23 | 3.01 | 5.9% | 9 | 99.1 |
| 546 | 544.98 | 21.08 | 3.9% | 9 | 99.8 |
| DHA | | | | | |
| 5.87 | 5.7 | 0.44 | 7.8% | 7 | 97.1 |
| 117 | 118.72 | 8.52 | 7.2% | 8 | 101.5 |
| 1,880 | 1,834.22 | 76.03 | 4.1% | 9 | 97.6 |

1. **WWARN / Incurred Plasma Samples**

Plasma samples spiked with AS and DHA provided by WWARN as part of their Quality Assurance /Proficiency testing programme were included with the batches. For both AS and DHA all 12 analyses measured would have provided a “satisfactory” result according to the WWARN scoring system.

1. **Patient’s Plasma AS and DHA Concentrations**

Plasma AS and DHA concentrations in malaria patients are listed at Table S3.

Table S3. Plasma AS and DHA concentrations (ng/mL).

| Patient Code | AS (ng/mL) | DHA (ng/mL) |
| --- | --- | --- |
| VAS01A | 23.05 | 1,012.4 |
| VAS01C | 41.19 | 856.55 |
| VAS02A | 36.49 | 1,547.35 |
| VAS03A | 70.09 | 668.18 |
| VAS04A | 227.60 | 823.60 |
| VAS05A | 30.36 | 215.11 |
| VAS02C | 36.59 | 74.36 |
| VAS06A | 51.08 | 235.33 |
| VAS03C | 153.41 | 1,379.69 |
| VAS08A | 46.00 | 731.16 |
| VAS09A | 20.50 | 163.16 |
| VAS10A | 11.00 | 1,697.36 |

- 1. **LC/MS/MS analysis of LUM and LUMm**

**1.1.1 LC/MS/MS Conditions**

Sample extracts were analysed on an AB Sciex 4000 QTRAP LC-MS/MS analyser (Applied Biosystems) preceded by a Prominence liquid chromatography system (Shimadzu), including a temperature-controlled autosampler.

- - 1. **Chromatographic Conditions**

LC-Column: Phenomenex Gemini-NX 5u C18 110A 50 mm x 2.0 mm I.D. 5 μm (Phenomenex, Torrance, CA, USA)

Pre-Column: Phenomenex Gemini-NX C18 4 mm x 2.0 mm I.D. 5 μm

Mobile Phase A: 2 mM Ammonium Acetate, 0.1% formic acid in Water

Mobile Phase B: 2 mM Ammonium Acetate, 0.1% formic acid in Methanol

Mobile Phase Flow: Gradient as follows:

| Time | Module | Events | Parameter |
| --- | --- | --- | --- |
| 0.60 | Pumps | Pump B Conc. | 70 |
| 0.90 | Pumps | Pump B Conc. | 95 |
| 1.10 | Pumps | Pump B Conc. | 95 |
| 1.20 | Pumps | Pump B Conc. | 70 |
| 3.00 | Pumps | Pump B Conc. | 70 |
| 3.10 | System Controller | Stop |  |

Column Oven: 40°C

Detector: ABSciex 4000 Qtrap LCMS Spectrophotometer

Injector Rinse Solution: 50% acetonitrile with 0.1% formic acid

Auto Sample Chamber Temp: 4-8°C

Regression: ABSciex Multiquant 2.1 MP4 integration with quadratic regression analysis with a weighting of 1/x^2^

- - 1. **MS Conditions**

Source conditions

| Curtain gas | 20 |
| --- | --- |
| CAD gas | Medium |
| IS (V) | 5,500 |
| Temp (˚C) | 600 |
| GS1 | 55 |
| GS2 | 50 |
| IHE | On |

Mass transitions

| Analyte | Q1  (Da) | Q3  (Da) | Time  (msec) | DP  (v) | EP  (v) | CE  (v) | CXP  (v) |
| --- | --- | --- | --- | --- | --- | --- | --- |
| LUM | 529.182 | 511.000 | 150 | 111 | 10 | 35 | 12 |
| LUMm | 474.166 | 456.000 | 150 | 86 | 10 | 27 | 24 |
| LUMa | 501.210 | 483.100 | 150 | 91 | 10 | 29 | 12 |

- - 1. **Testing of Drug-Free Plasma (DFP) for Ion Suppression**

Six drug-free citrate phosphate dextrose anticoagulated human plasma (DFPs) were tested for matrix effect (ion suppression) to identify drug-free plasma that showed no identifiable effect on drug quantification. This was done by infusing a low concentration of LUM, LUMm and LUMa while injecting mobile phase extracts from the six DFPs. None of the plasmas showed ion suppression and one of these, was used to prepare calibrators and quality control (QC) samples.

**1.1.5 Preparation of Calibration and Internal Standards**

- LUM, Source WWARN, Alsa Chim, Sample ID RMLUM20140828-08, Lot # CM-ALS-10-004. MW 528.94 g/mol.
- LUMm, (N-Desbutyl-Lumefantrine Oxalate salt) WWARN, Alsa Chim, Sample ID RMLUMm20111209-09, Lot # JFH-ALS-11-034-P1. MW Salt 562.86 g/mol. MW Base 472.86 g/mol. Ratio 0.8401.
- LUMa (LUM analog, internal standard), WWARN, Alsa Chim, Sample ID RMLUMa20130827-01, Lot # BD-ALS-11-004-P1. MW Salt 590.92 g/mol. MW Base 500.92 g/mol. Ratio 0.848.
- Quantities of LUM, LUMm and LUMa were weighed and the amount of base compound calculated. The appropriate volume of methanol was added to produce a stock solution of 1 mg/mL base. Intermediate solutions were prepared in 50% methanol as shown below. All stock solutions were prepared on ice and kept at -80^o^ C until required.
- Preparation of intermediate solutions.

| Intermediate solution | Concentration (µg/mL) |
| --- | --- |
| IS_1_ | 100 |
| IS_2_ | 10 |
| IS_3_ | 1 |
| IS_4_ | 0.1 |

- Intermediate solutions for LUM and LUMm were prepared separately as 100 µg/mL, 10 µg/mL, 1 µg/mL and 100 ng/mL solutions by the sequential 10-fold dilution of the 1 mg/mL stocks using 100 µL diluted into 900 µL of 50% methanol. Solutions were stored at -80^o^C.
- Calibration standards were prepared volumetrically in DFP on ice as indicated below. The calibration standards were aliquoted and stored at

-80^o^C.

- Preparation of Calibration Standards for LUM and LUMm.

| STD | LUM Final  Conc.  (ng/mL) | Volume LUM Interm. Soln.  (μL) | LUM Interm. Soln.  (µg/mL) | LUMm Final  Conc.  (ng/mL) | Volume LUMm Interm.Soln  (μL) | LUMm Interm. Soln. (µg/mL) | Volume of blank DFP (μL) |
| --- | --- | --- | --- | --- | --- | --- | --- |
| S1 | 2 | 80 | 0.1 | 1 | 40 | 0.1 | 3,880 |
| S2 | 5 | 20 | 1 | 2 | 8 | 1 | 3,972 |
| S3 | 30 | 12 | 10 | 5 | 20 | 1 | 3,968 |
| S4 | 200 | 80 | 10 | 30 | 12 | 10 | 3,908 |
| S5 | 1,000 | 40 | 100 | 200 | 8 | 100 | 3,952 |
| S6 | 3,000 | 120 | 100 | 1,000 | 40 | 100 | 3,840 |

- LUMa was diluted with methanol to produce a stock solution of 1 mg/mL base. This was further diluted in 50% methanol to produce an intermediate stock solution of 1 µg/mL and stored at 4^o^C. A 1/100 dilution of this intermediate solution was made just before use to attain a final internal standard working solution of 10 ng/mL.

**1.1.6 Preparation of QC Plasma Samples**

Quality control (QC) samples containing LUM and LUMm were prepared in DFP on ice covering a low, mid and high range .The QC samples were analysed in duplicate with each analytical batch in an identical manner to the calibration and unknown plasma samples. A quantification run was considered invalid if more than two QC samples observed values deviated by > ±15% of their calculated value, or if two QC samples from the same group, low, mid or high, deviated by > ±15%.

- Preparation of Quality Control Samples.

| QC | LUM Final  Conc.  (ng/mL) | Volume LUM Interm Soln.  (μL) | LUM Interm Soln.  (µg/mL) | LUMm Final  Conc.  (ng/mL) | Volume  LUMm Interm Soln.  (μL) | LUMm Interm Soln.  (µg/mL) | Volume of blank DFP  (μL) |
| --- | --- | --- | --- | --- | --- | --- | --- |
| QCH | 2,000 | 80 | 100 | 500 | 20 | 100 | 3,900 |
| QCM | 100 | 40 | 10 | 50 | 20 | 10 | 3,940 |
| QCL | 10 | 40 | 1 | 4 | 16 | 1 | 3,944 |

**1.1.7 Sample Preparation**

- Subject plasma samples were thawed in a room temperature circulating water bath and vortex-mixed prior to analysis.
- Calibrators and QCs were prepared by spiking LUM and LUMm in DFP (50 µL) with acetonitrile containing internal standard (10 ng/mL LUMa in acetonitrile; 200 µL), or blank acetonitrile (200 µL) for the double blank. Calibrators and blanks were then treated as unknowns.
- 50 µL of 0.2 M ZnSO4 was added to pre-labelled 1.5 mL polypropylene microfuge tubes.
- Subject or spiked plasma (50 µL) was added.
- 200 µL of internal standard solution was added.
- The tubes were mixed by vortexing for 1 min at maximum vortex setting and then centrifuged (20,817 x *g*) for 5 min at 4°C.
- The organic layer (100 µL) was subsequently transferred to a 96 well polypropylene microtitre assay plate.
- 10 µL was injected on column.

**1.1.8 Quantification**

Chromatograms were integrated and processed using Applied Biosystems software (Analyst V1.4.2). Concentrations of LUM and LUMm in subject plasma samples and quality controls were calculated from regression curves of peak area ratios (LUM:LUMa and LUMm:LUMa) versus analyte concentration using Multiquant 2.1 software. A weighting of (1/X^2^) was used to generate a quadratic regression curve.

**1.1.9 QA/QC**

Paired samples of each QC blood sample were run with each batch.

- 1. **Chromatograms**

**2.1.1 LUM and LUMm**

Typical chromatograms of LUM and LUMm in plasma are shown in Figures S1 to S6. The retention times for LUM, LUMm and LUMa were approximately at 1.5, 1.2 and 0.9 min, respectively.

Figure S1. Drug-free plasma (DFP) for LUM and LUMm analysis

| 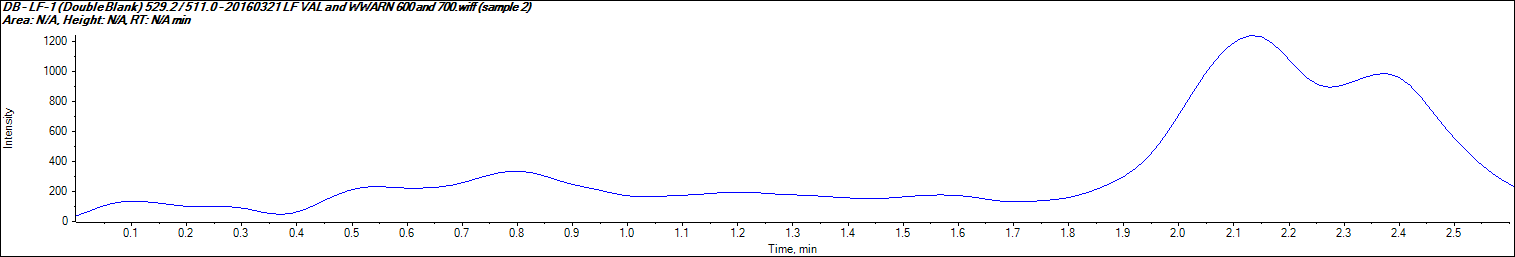 | 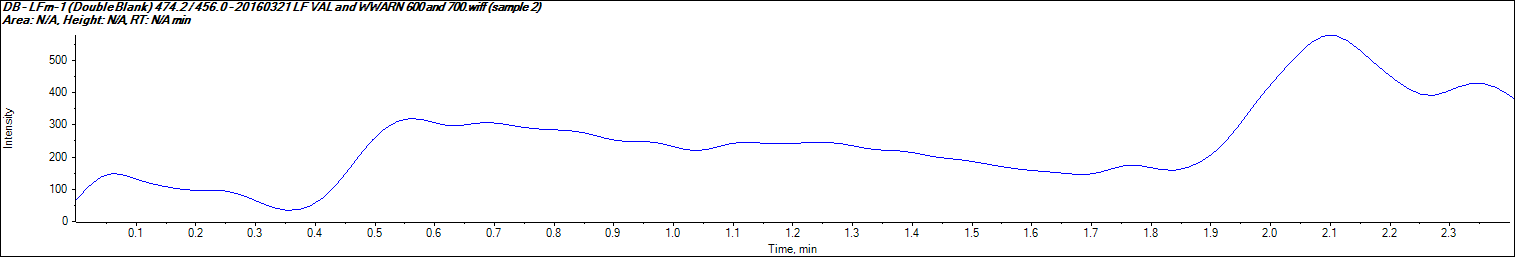 |
| --- | --- |

Figure S2. Low-range QC plasma nominal concentrations of LUM (10 ng/mL) and LUMm (3.2 ng/mL)

| 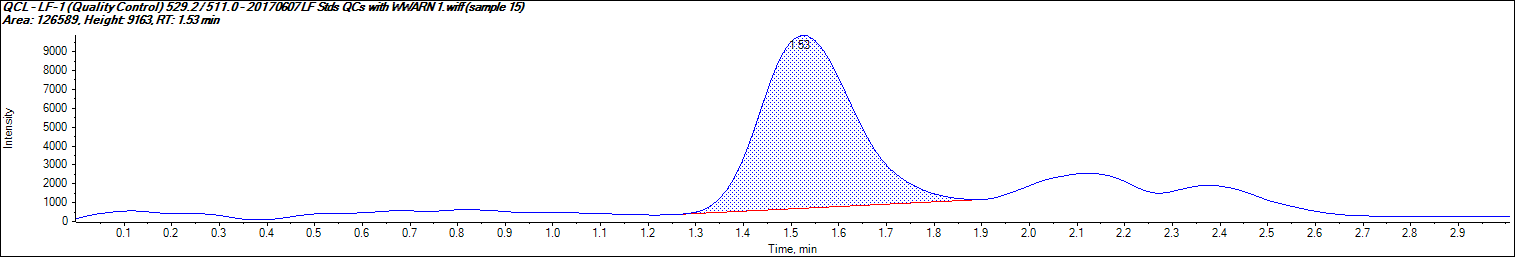 | 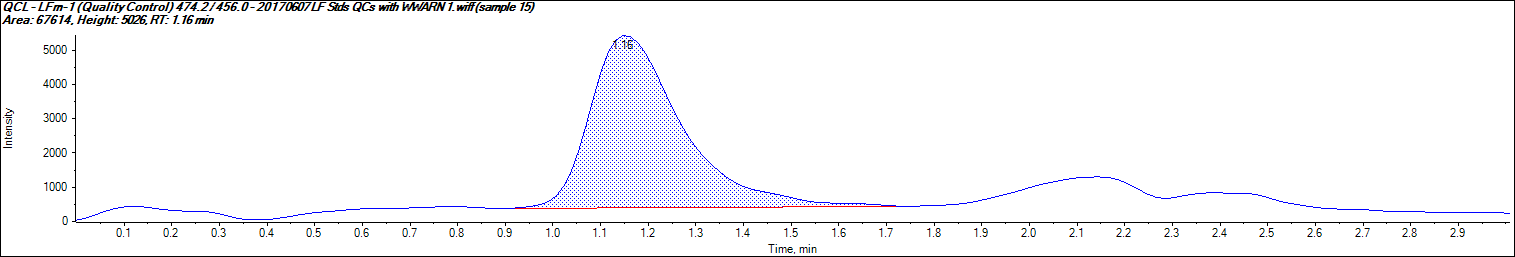 |
| --- | --- |

Figure S3. Spiked DFP containing nominal concentrations of LUM (2 ng/mL) and LUMm (1 ng/mL)

| 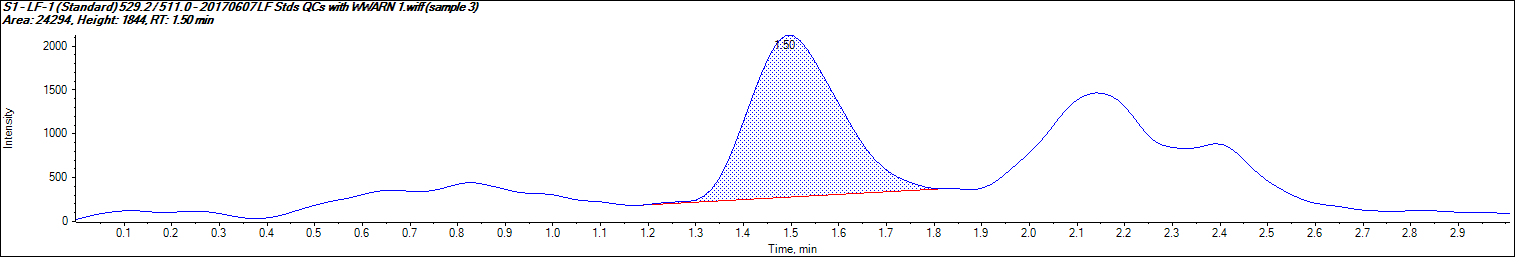 | 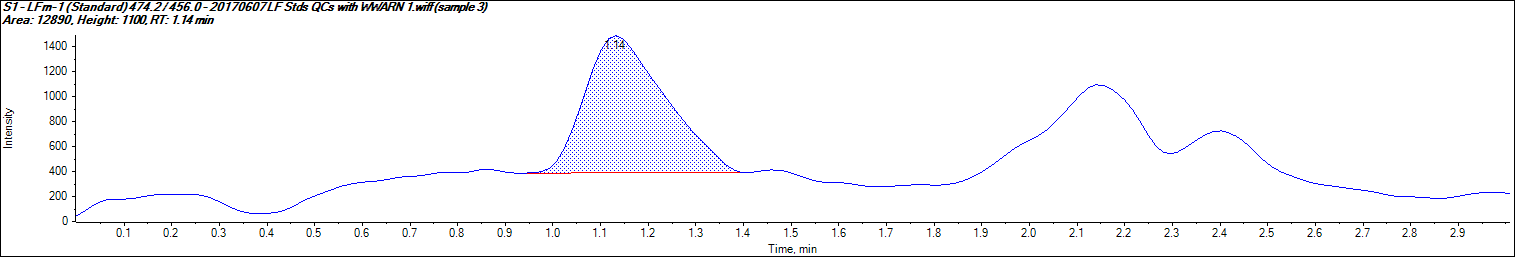 |
| --- | --- |


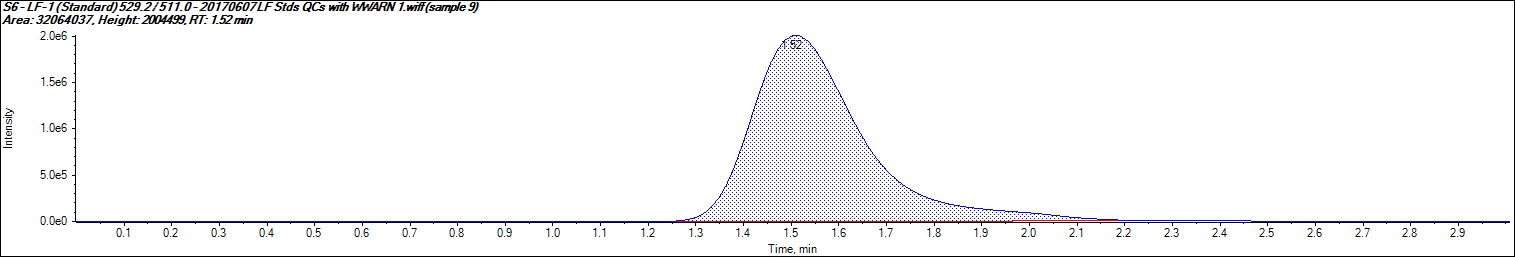
Figure S4. Spiked DFP containing nominal concentrations of LUM (3,000 ng/mL) and LUMm (1,000 ng/mL)


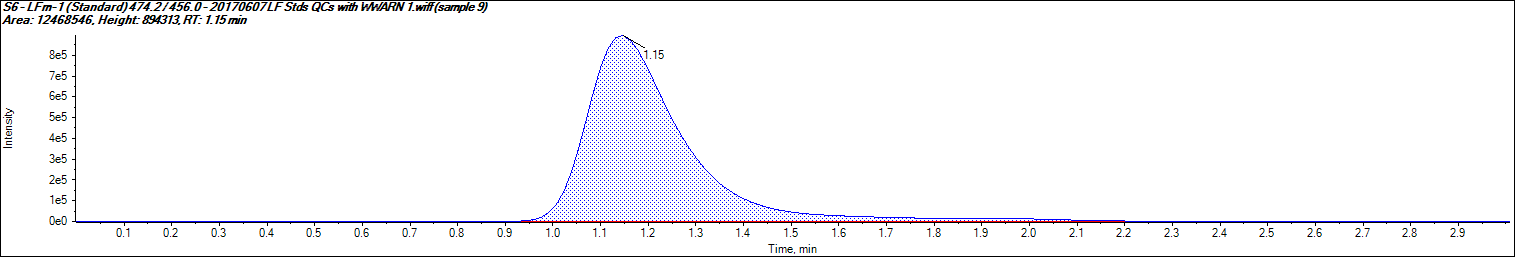


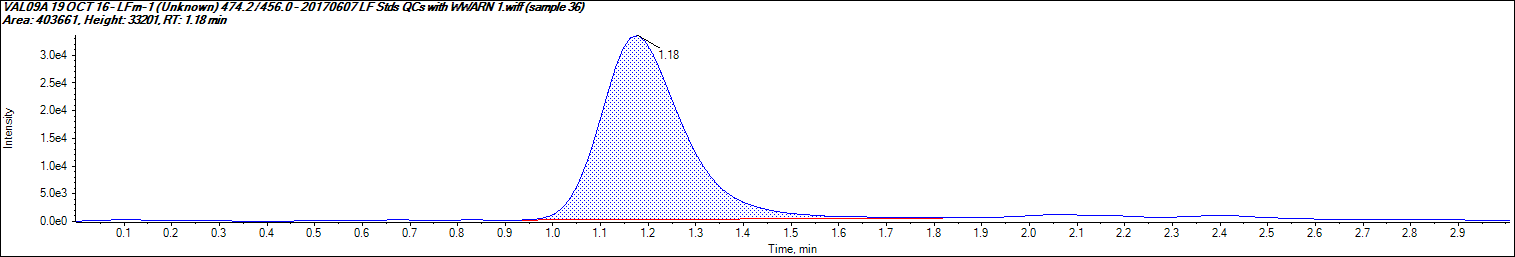

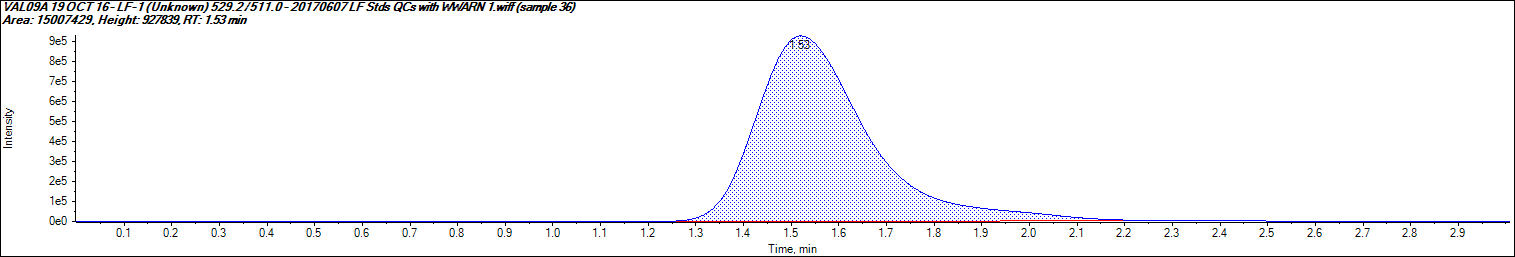
Figure S5. Plasma sample from patient VAL09A (LUM 1229 ng/mL; LUMm 20.1 ng/mL)


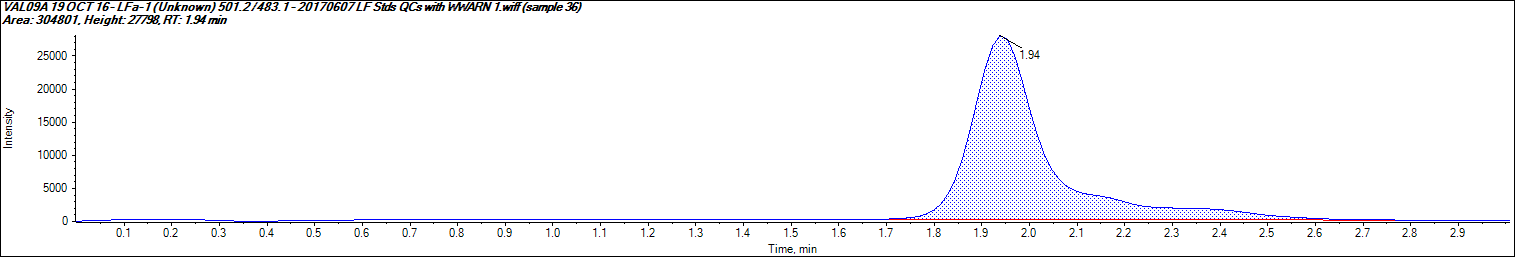
Figure S6. LUMa in extracted plasma sample for patient VAL09A

**2.2 Method Validation Plasma Samples**

**2.2.1 LUM and LUMm**

- - - 1. **Accuracy and Precision**

Calibration standards of plasma LUM and LUMm concentrations from 2 to 3,000 and 1 and 1,000 ng/mL, respectively, were analysed on different days (Table S1 for LUM and Table S2 for LUMm). Good linearity was obtained over this range, with good precision and accuracy.

Table S1. Interday Accuracy and Precision of LUM assay

| Nominal Conc. (ng/mL) | Mean (ng/mL) | SD (ng/mL) | CV  (%) | n | Mean Accuracy (%) |
| --- | --- | --- | --- | --- | --- |
| 2 | 2.00 | 0.06 | 3.23 | 13 | 100.00 |
| 5 | 4.98 | 0.18 | 3.61 | 9 | 99.62 |
| 30 | 30.42 | 1.42 | 4.66 | 9 | 101.39 |
| 200 | 199.64 | 5.27 | 2.64 | 9 | 99.82 |
| 1,000 | 985.62 | 42.15 | 4.28 | 9 | 98.56 |
| 3,000 | 3015.05 | 131.98 | 4.38 | 12 | 100.50 |

Table S2. Interday Accuracy and Precision of LUMm assay

| Nominal Conc. (ng/mL) | Mean (ng/mL) | SD (ng/mL) | CV  (%) | n | Mean Accuracy (%) |
| --- | --- | --- | --- | --- | --- |
|  |  |  |  |  |  |
| 1 | 0.99 | 0.03 | 3.27 | 13 | 99.31 |
| 2 | 2.01 | 0.08 | 4.12 | 9 | 100.28 |
| 5 | 5.17 | 0.27 | 5.17 | 9 | 103.38 |
| 30 | 30.89 | 0.93 | 3.02 | 9 | 102.97 |
| 200 | 184.57 | 8.86 | 4.80 | 9 | 92.29 |
| 1,000 | 1,023.79 | 61.39 | 6.00 | 12 | 102.38 |

- For LUM the lower limit of quantification (LLOQ) using 50 µL of plasma was 2 ng/mL, with a coefficient of variation (CV) of 3.2 % and an accuracy of 100%.
- For LUMm the lower limit of quantification (LLOQ) using 50 µL of plasma was 1 ng/mL, with a CV of 3.3% and an accuracy of 99.3%.
- A quadratic regression equation *y = ax^2^ + bx+ c* where *x* is the amount of drug and *y* is the peak area ratio, was used to determine the concentrations of unknowns and QC samples. A typical regression equation of a calibration curve was:

LUM: a = -3. 69806x10^-6^, b = 0.04007, c = -0.00545, with r = 0.99923

LUMm: a = -2.33131^-5^, b = 0.07254 c = -0.00848, with r = 0.99384.

- - - 1. **QC Plasma Samples**

Paired QC samples for low, mid and high concentrations from were included with each analytical batch. Results of accuracy and precision of QC samples (mid-low-high concentrations) that fell within acceptance criteria are shown in Table S3 and Table S4.

Table S3. Accuracy and Precision of Plasma LUM QC samples

| Nominal Conc. (ng/mL) | Mean (ng/mL) | SD (ng/mL) | CV  (%) | n | Mean Accuracy (%) |
| --- | --- | --- | --- | --- | --- |
|  |  |  |  |  |  |
| 10 | 9.76 | 0.71 | 7.26 | 11 | 97.59 |
| 100 | 104.76 | 8.24 | 7.86 | 11 | 104.76 |
| 2,000 | 2,053.15 | 185.06 | 9.01 | 11 | 102.66 |

Table S4. Accuracy and Precision of Plasma LUMm QC samples

| Nominal Conc. (ng/mL) | Mean (ng/mL) | SD (ng/mL) | CV  (%) | n | Mean Accuracy (%) |
| --- | --- | --- | --- | --- | --- |
|  |  |  |  |  |  |
| 4 | 4.21 | 0.16 | 3.80 | 11 | 105.18 |
| 50 | 52.30 | 2.84 | 5.44 | 11 | 104.61 |
| 500 | 490.05 | 38.38 | 7.83 | 11 | 98.01 |

- - - 1. **WWARN / Incurred Plasma Samples**

Plasma samples spiked with LUM and LUMm provided by WWARN as part of their Quality Assurance /Proficiency testing programme were included with the batches. For both LUM and LUMm all 14 analyses measured would have provided a “satisfactory” result according to the WWARN scoring system.

- - - 1. **Patient’s Plasma LUM and LUMm Concentrations**

Plasma LUM and LUMm concentrations in malaria patients are listed in Table S5.

Table S5. Plasma LUM and LUMm concentrations (ng/mL).

| Patient Code | LUM | LUMm |
| --- | --- | --- |
| VAL01C | 887.9 | 25.8 |
| VAL01A | 988.0 | 12.4 |
| VAL02A | 803.7 | 14.6 |
| VAL02C | 194.0 | 5.2 |
| VAL03A | 276.6 | 6.0 |
| VAL03C | 474.7 | 22.1 |
| VAL04A | 535.4 | 13.5 |
| VAL05A | 958.9 | 16.1 |
| VAL06A | 590.3 | 14.4 |
| VAL07A | 637.3 | 13.7 |
| VAL08A | 763.7 | 12.5 |
| VAL04C | 235.3 | 5.6 |
| VAL09A | 505.1 | 17.8 |
| VAL10A | 774.6 | 15.3 |

- 1. **LC/MS/MS analysis of CQ and CQM**

**1.1.1 LC/MS/MS Conditions**

Sample extracts were analysed on an AB Sciex 4000 QTRAP LC-MS/MS analyser (Applied Biosystems) preceded by a Prominence liquid chromatography system (Shimadzu), including a temperature-controlled autosampler.

- - 1. **Chromatographic conditions**

LC-Columns: Phenomenex PFP-2, 5 µm column, 50 mm x 4.6 mm. (Phenomenex, USA)

Pre-Column: Phenomenex PFP2, 5 µm, 4 x 2 mm I.D. guard column.

Mobile Phase A: A: 2 mM Perfluoro-octanoic Acid (PFOA).

Mobile Phase B: Acetonitrile (15:85 A:B)

Pump: Flow rate: A: 0.25 mL/min

B: 0.25 mL/min

Total: 0.5 mL/min

Column Oven Temp: 30°C.

Detector: ABSciex 4000 Qtrap LCMS Spectrophotometer

Injector: Rinse solution 50% Acetonitrile/ 1% Formic Acid

Auto Sample Chamber Temp: 4-8°C.

Regression: ABSciex Multiquant 2.1 MP4 integration with quadratic regression analysis with a weighting of 1/x^2^

- - 1. **MS Conditions**

Source conditions

| Curtain gas | 50 |
| --- | --- |
| CAD gas | Medium |
| IS (V) | 3,500 |
| Temp (˚C) | 500 |
| GS1 | 65 |
| GS2 | 60 |
| IHE | On |

Mass Transitions:

| Analyte | Q1  (Da) | Q3  (Da) | Time  (msec) | DP  (v) | EP  (v) | CE  (v) | CXP  (v) |
| --- | --- | --- | --- | --- | --- | --- | --- |
| CQ-1 | 320.2 | 247.1 | 150 | 106 | 10 | 29 | 26 |
| CQm-2 | 292.2 | 114.3 | 150 | 91 | 10 | 29 | 10 |
| CQa-1 | 334.2 | 261.1 | 150 | 96 | 10 | 31 | 24 |
|  |  |  |  |  |  |  |  |

- - 1. **Testing of Drug-Free Blood (DFB) for Ion Suppression**

Six DFBs were tested for matrix effect (ion suppression) to identify drug-free blood that showed no identifiable effect on drug quantification. This was done by infusing a low concentration of CQ, CQm and CQa while injecting mobile phase extracts from the six DFBs. None of the bloods showed ion suppression and one of these, was used to prepare calibrators and quality control (QC) samples.

- - 1. **Preparation of Calibration, Internal Standards and QCs**
- Chloroquine Analog (CQa), WWARN Reference Compound Stock Solution: between 1-2 mg of CQa was weighed and dissolved in a volume of 50% MeOH (DIL1) to produce a concentration of 1 mg/mL.
- Intermediate solution 1 (CQ-IS1) a 1:10 dilution of stock in DIL1was made to produce a concentration of 100 μg/mL. Intermediate solution 2 (CQ-IS2) a 1:100 dilution of IS1 was made to produce a concentration of 1 μg/mL.
- Working Solution (CQ-Internal Std): 50 μL of CQ-IS2 was added to 49.95 mL of 50% Acetonitrile to produce a final concentration of 1 ng/mL (stable 1 week at 4°C).
- Chloroquine diphosphate (CQ) WWARN Reference Compound
  - MW (salt) = 516; MW (base) = 320
  - Conversion Factor = 1.61 (i.e. 1.61 mg salt = 1.0 mg base)
  - Stock Solution: A quantity of CQ salt was weighed and divided by the conversion factor to determine the quantity of base. This was dissolved in an *w/v* of DIL1 to give a concentration of 1 mg/mL.
- Desethyl-chloroquine diphosphate (CQm) WWARN Reference Compound
  - MW (salt) = 488; MW (base) = 292
  - Conversion Factor = 1.67 (i.e. 1.67 mg salt = 1.0 mg base)
  - Stock Solution: A quantity of CQm salt was weighed and divided by the conversion factor to determine the quantity of base. This was dissolved in a *w/v* of DIL1 to give a concentration of 1 mg/mL.
- Combination CQ/CQm Intermediate Solution: 100 µL aliquots of the CQ and CQm stock were further diluted in 800 µL DIL1 to prepare a combined intermediate solution with a concentration of 100 µg/mL of both CQ and CQm (CQ/CQm-IS1). All stocks and solutions were stored at -80°C.
  - - Working Solutions: A series of working solutions was prepared as follows:

| WS 2000 | 100 µL CQ/CQm-IS1 + 4.9 mL  Drug Free Matrix (DFM) | = | 2,000 ng/mL |
| --- | --- | --- | --- |
| WS 1000 | 1:2 Dilution of WS1000 in DFM | = | 1,000 ng/mL |
| WS 500 | 1:2 Dilution of WS1000 in DFM | = | 500 ng/mL |
| WS 100 | 1:10 Dilution of WS1000 in DFM | = | 100 ng/mL |
| WS 50 | 1:1 Dilution of WS500 in DFM | = | 50 ng/mL |
| WS 10 | 1:10 Dilution of WS100 in DFM | = | 10 ng/mL |
| WS 5 | 1:10 Dilution of WS50 in DFM | = | 5 ng/mL |
| WS 1 | 1:10 Dilution of WS10 in DFM | = | 1 ng/mL |
| WS 0.5 | 1:10 Dilution of WS5 in DFM | = | 0.5 ng/mL |
| QCH | 60 µL CQ/CQm-IS1 + 9.94 mL DFM | = | 600 ng/mL |
| QCM | 1:3 Dilution of QCH in DFM | = | 200 ng/mL |
| QCL | 1:40 Dilution of QCM in DFM | = | 5 ng/mL |

- Contents were mixed and allowed to stand for 30 min at 4ºC. 150 µL aliquots were then dispensed into 1.8 mL polypropylene micro centrifuge tubes and frozen at -80°C.
- Quality control (QC) samples containing CQ and CQm were prepared in DFB on ice covering a low, mid and high range .The QC samples were analysed in duplicate with each analytical batch in an identical manner to the calibration and unknown blood samples. A quantification run was considered invalid if more than two QC samples observed values deviated by > ±15% of their calculated value, or if two QC samples from the same group, low, mid or high, deviated by > ±15%.
  - 1. **Sample Preparation**
- Subject blood samples were thawed in a room temperature circulating water bath and vortex-mixed prior to analysis.
- 25 μL of CQa to 50 μL of sample was added to polypropylene microcentrifuge tubes and samples moved to a fume hood.
- 100 μL 30% ammonium solution was added to each tube followed by 1,000 μL Methyl-*tert*-Butyl ether.
- Samples were mixed on an Intellimix combination vortex/rotator for 10 min at room temperature on a setting of 99 rpm and then centrifuged at 20,000 x *g* for 15 min at 4°C.
- Samples were moved to a fume hood and 800 μL of supernatant transferred to a clean micro centrifuge tube.
- Samples were evaporated under a stream of instrument grade air at 40°C followed by reconstitution with 150 μL of 50:50 Part A:Part B mobile phase.
- 5 µL was injected on column.
  - 1. **Quantification**

Chromatograms were integrated and processed using Applied Biosystems software (Analyst V1.4.2). Concentrations of CQ and CQm in subject blood samples and quality controls were calculated from regression curves of peak area ratios (CQ:CQa and CQm:CQa) versus analyte concentration using Multiquant 2.1 software. A weighting of (1/X^2^) was used to generate a quadratic regression curve.

- - 1. **QA/QC**

Paired samples of each QC blood sample were run with each batch.

**2.1 Chromatograms**

**2.1.1 CQ and CQm**

Typical chromatograms of CQ and CQm in blood are shown in Figures S1 to S6. The retention times for CQ, CQm and CQa were approximately at 0.84, 0.78 and 0.89 min, respectively.

Figure S1. Drug-free blood sample for CQ and CQm analysis

| 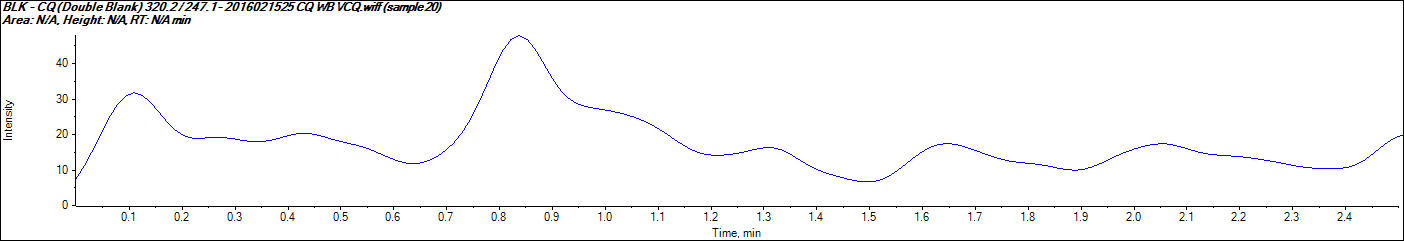 | 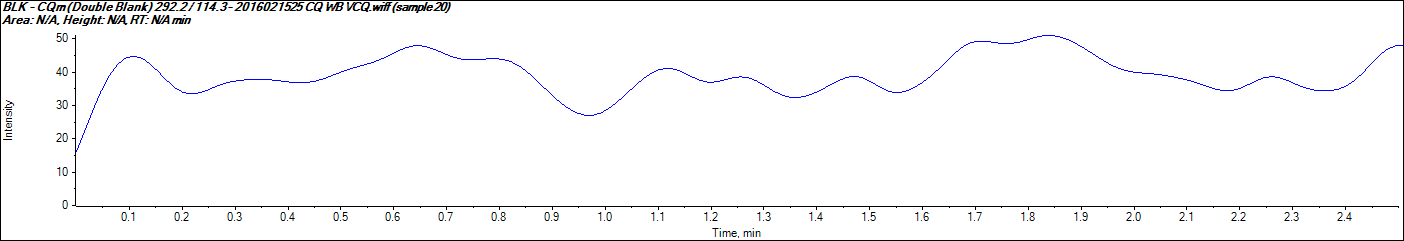 |
| --- | --- |

Figure S2. Low-range QC blood containing nominal concentrations of CQ and CQm (5 ng/mL)

| 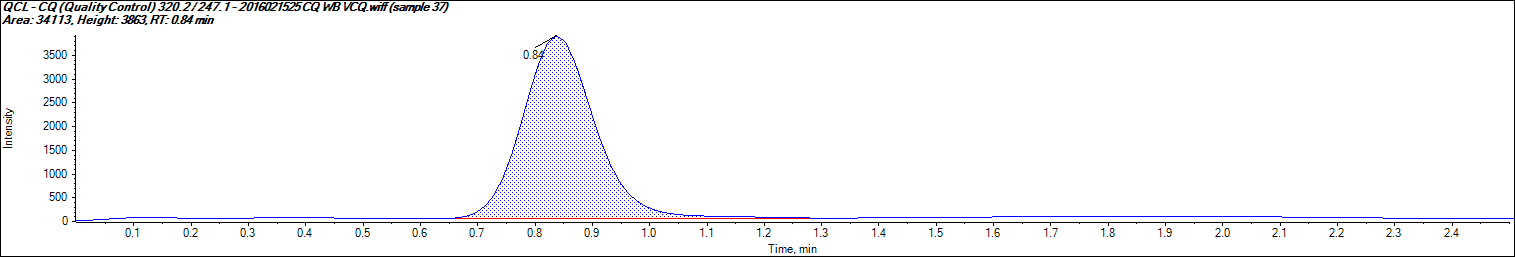 | 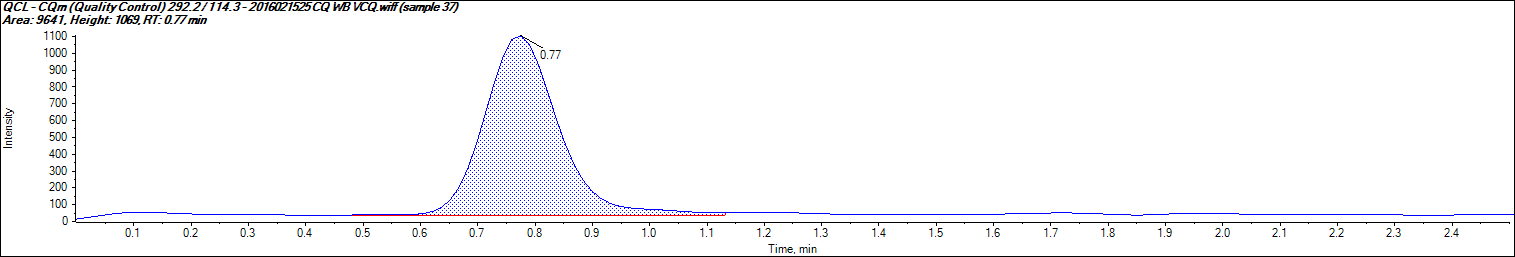 |
| --- | --- |

Figure S3. Spiked DFB containing nominal concentrations of CQ and CQm (0.5 ng/mL)

| 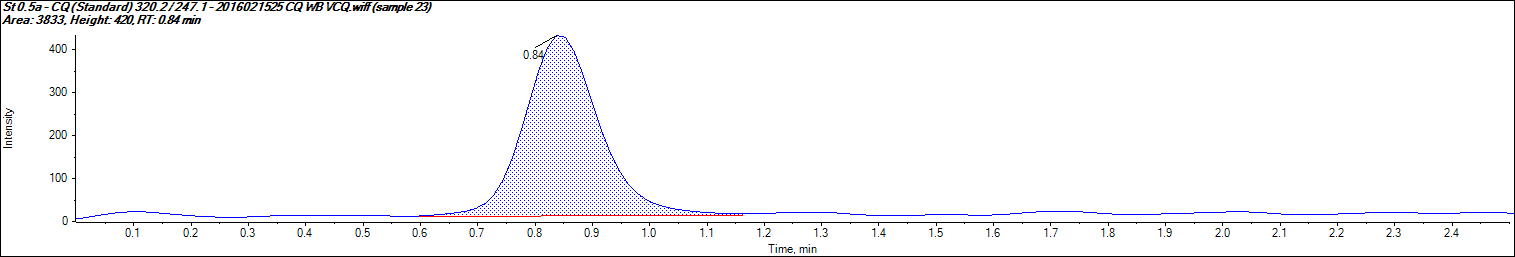 | 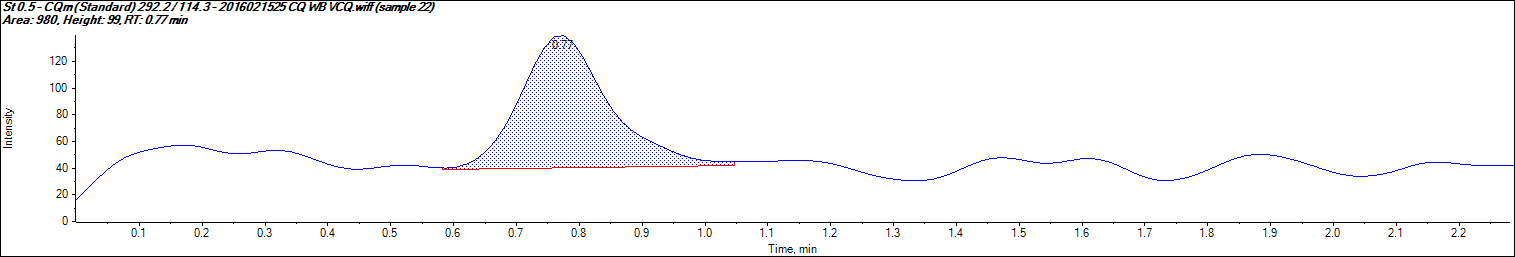 |
| --- | --- |

Figure S4. Spiked DFB containing nominal concentrations of CQ and CQm (1,000 ng/mL)

| 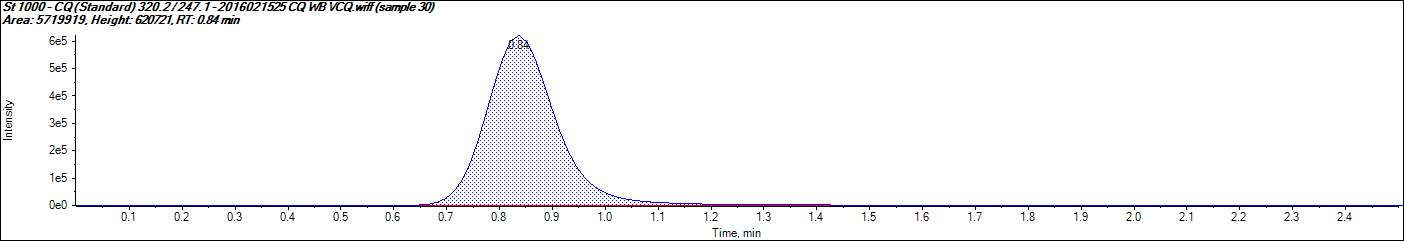 | 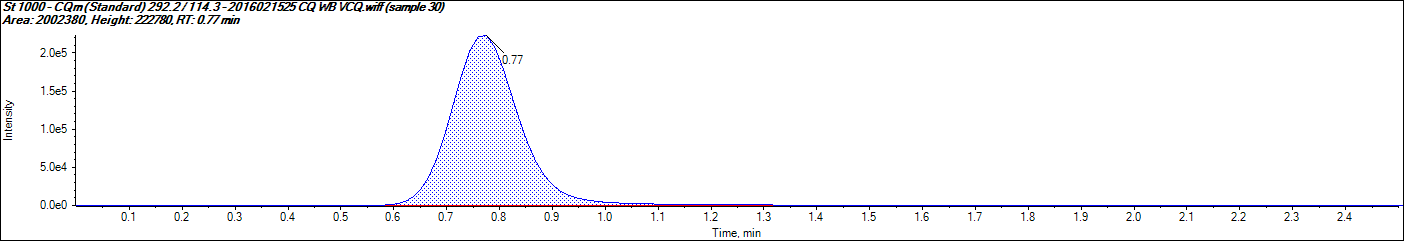 |
| --- | --- |

Figure S5. Blood sample from patient VCQ08 (CQ 34.92 ng/mL; CQm 47.16 ng/mL) at day 28

| 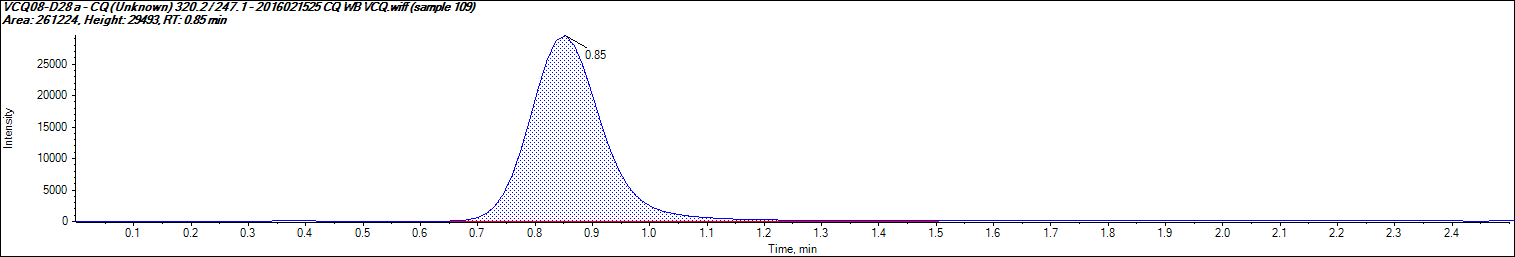 | 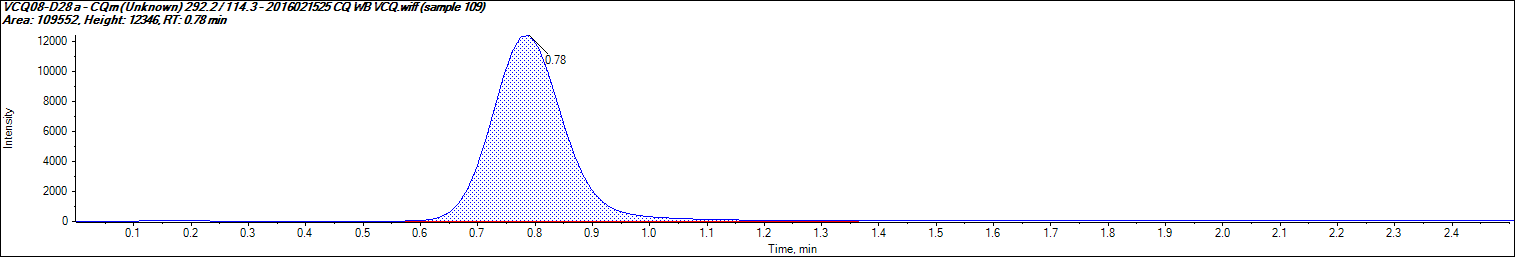 |
| --- | --- |

Figure S6. CQa in extracted blood sample for patient VCQ08

| 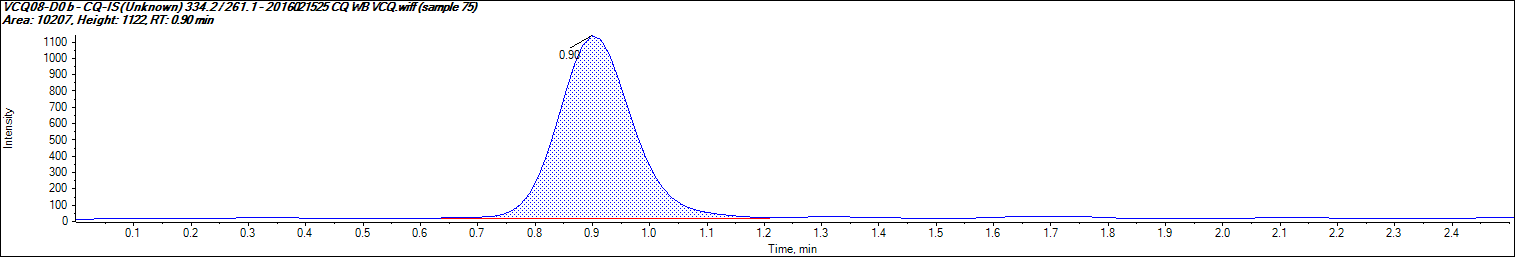 |  |
| --- | --- |

**2.2. Method Validation Blood Samples**

- - 1. **CQ and CQm**
       1. **Accuracy and Precision**

Calibration standards of blood CQ and CQm concentrations from 0.5 to 1,000 ng/mL were analysed on different days (Table S1). Good linearity was obtained over this range, with good precision and accuracy.

Table S1. Inter-day Accuracy and Precision of Blood CQ/CQm assay.

| Nominal Conc. (ng/mL) | Mean (ng/mL) | SD (ng/mL) | CV  (%) | n | Mean  Accuracy (%) |
| --- | --- | --- | --- | --- | --- |
| CQ | | | | | |
| 0.5 | 0.51 | 0.05 | 9.1% | 8 | 101.3 |
| 1 | 0.96 | 0.06 | 6.6% | 8 | 95.8 |
| 5 | 4.87 | 0.38 | 7.8% | 7 | 97.4 |
| 10 | 9.42 | 0.70 | 7.4% | 8 | 94.2 |
| 50 | 52.32 | 3.08 | 5.9% | 8 | 104.6 |
| 100 | 102.92 | 4.42 | 4.3% | 9 | 102.9 |
| 500 | 518.54 | 20.37 | 3.9% | 7 | 103.7 |
| 1,000 | 956.36 | 52.19 | 5.5% | 8 | 95.6 |
| CQm | | | | | |
| 0.5 | 0.49 | 0.03 | 6.8% | 8 | 98.1 |
| 1 | 1.00 | 0.09 | 9.2% | 7 | 99.8 |
| 5 | 5.18 | 0.33 | 6.3% | 9 | 103.7 |
| 10 | 9.72 | 0.51 | 5.2% | 9 | 97.2 |
| 50 | 51.28 | 2.71 | 5.3% | 8 | 102.6 |
| 100 | 98.50 | 3.87 | 3.9% | 8 | 98.5 |
| 500 | 497.73 | 29.58 | 5.9% | 9 | 99.5 |
| 1,000 | 1,005.52 | 79.59 | 7.9% | 9 | 100.6 |

- For CQ the lower limit of quantification (LLOQ) using 50 µL of blood was 0.5 ng/mL, with a coefficient of variation (CV) of 9.1% and an accuracy of 101.3%.
- For CQm the lower limit of quantification (LLOQ) using 50 µL of blood was 0.5 ng/mL, with a CV of 6.8% and an accuracy of 98.1%.
- A quadratic regression equation *y = ax^2^ + bx+ c* where *x* is the amount of drug and *y* is the peak area ratio, was used to determine the concentrations of unknowns and QC samples. A typical regression equation of a calibration curve was:

CQ: a = -2.49669x10^-5^, b = 0.25590, c = 0.05682, with r = 0.99905

CQm: a = -1.08960 x10^-5^, b = 0.17796, c = 0.04845, with r = 0.99956

- - - 1. **QC Blood Samples**

Paired QC samples for low, mid and high concentrations from were included with each analytical batch. Results of accuracy and precision of QC samples (mid-low-high concentrations) that fell within acceptance criteria are shown in Table S2.

TableS 2. Accuracy and Precision of Blood CQ and CQm QC samples

| Nominal Conc. (ng/mL) | Mean (ng/mL) | SD (ng/mL) | CV  (%) | n | Mean  Accuracy (%) |
| --- | --- | --- | --- | --- | --- |
| CQ | | | | | |
| 5 | 5.00 | 0.21 | 4.20% | 6 | 100.03 |
| 200 | 207.49 | 5.77 | 2.78% | 6 | 103.75 |
| 600 | 609.25 | 24.26 | 3.98% | 6 | 101.54 |
| CQm | | | | | |
| 5 | 4.91 | 0.26 | 5.39% | 6 | 98.19 |
| 200 | 207.39 | 6.45 | 3.11% | 6 | 103.70 |
| 600 | 614.16 | 27.65 | 4.50% | 6 | 102.36 |

- - - 1. **WWARN / Incurred Plasma Samples**

WWARN does not have whole blood samples spiked with CQ and CQm as part of their Quality Assurance /Proficiency testing.

- - - 1. **Patient’s Blood CQ and CQm Concentrations**

Blood concentrations of CQ and CQm in malaria patients are listed in Table S3.

Table S3. Blood CQ and CQm concentrations (ng/mL) pre-dosing (day 0) and 28 days post dosing

| Patient code | CQ (ng/mL) | CQm (ng/mL) | Composite (ng/mL) |
| --- | --- | --- | --- |
| VCQ01 D0 | BLQ | BLQ | BLQ |
| VCQ02 D0 | BLQ | BLQ | BLQ |
| VCQ03 D0 | BLQ | BLQ | BLQ |
| VCQ04 D0 | BLQ | BLQ | BLQ |
| VCQ05 D0 | BLQ | BLQ | BLQ |
| VCQ06 D0 | BLQ | BLQ | BLQ |
| VCQ07 D0 | BLQ | BLQ | BLQ |
| VCQ08 D0 | 2.09 | 1.20 | 3.29 |
| VCQ09 D0 | BLQ | BLQ | BLQ |
| VCQ010 D0 | BLQ | BLQ | BLQ |
| VCQ011 D0 | BLQ | BLQ | BLQ |
| VCQ012 D0 | BLQ | BLQ | BLQ |
| VCQ13 D0 | BLQ | BLQ | BLQ |
| VCQ14 D0 | BLQ | BLQ | BLQ |
| VCQ15 D0 | BLQ | BLQ | BLQ |
| VCQ16 D0 | BLQ | BLQ | BLQ |
|  |  |  |  |
| VCQ01 D28 | 5.19 | 7.50 | 12.70 |
| VCQ02 D28 | 16.72 | 24.73 | 41.44 |
| VCQ03 D28 | 4.59 | 8.76 | 13.36 |
| VCQ05 D28 | 7.05 | 17.02 | 24.07 |
| VCQ06 D28 | 22.76 | 28.86 | 51.62 |
| VCQ07 D28 | 14.72 | 14.29 | 29.01 |
| VCQ08 D28 | 31.78 | 41.62 | 73.40 |
| VCQ09 D28 | 8.55 | 14.85 | 23.40 |
| VCQ010 D28 | 13.76 | 25.42 | 39.19 |
| VCQ011 D28 | 24.75 | 32.83 | 57.58 |
| VCQ012 D28 | 29.28 | 38.55 | 67.83 |
| VCQ13 D28 | 44.64 | 31.39 | 76.03 |
| VCQ14 D28 | 34.32 | 31.67 | 66.00 |
| VCQ15 D28 | 42.31 | 25.83 | 68.14 |
| VCQ16 D28 | 13.15 | 31.50 | 44.64 |

BLQ: Below the Limit of Quantification
