# Supplementary material for: Susceptibility of Plasmodium falciparum to artemisinins and Plasmodium vivax to chloroquine in Phuoc Chien Commune, Ninh Thuan Province, south-central Vietnam
Source: Malar J. 2019 Jan 17;18:10. doi: 10.1186/s12936-019-2640-2 (PMC6335800; doi:10.1186/s12936-019-2640-2)
Supplement: Supplementary file 3 — Additional file 3: Table S1. Adverse events before and after treatment of Vietnamese patients with AS + DHA–PPQ or AM–LUM for uncomplicated falciparum malaria. [file 12936_2019_2640_MOESM3_ESM.docx]

**Additional file 3: Table S1.** Adverse events before and after treatment of Vietnamese patients with AS+DHA-PPQ or AM-LUM for uncomplicated falciparum malaria

| **AS+DHA-PPQ** | **Before**  **Treatment (D0)** | **D1-24 h** | **D2-48 h** | **D3-72 h** |
| --- | --- | --- | --- | --- |
| Rigors/Chills | 92.3% (12/13) | 7.7% (1/13) | NR | NR |
| Sweating | 92.3% (12/13) | 7.7% (1/13) | NR | NR |
| Headache | 100.0% (13/13) | 84.6%(11/13) | 61.5% (8/13) | 23.1% (3/13) |
| Nausea | 23.1% (3/13) | NR | NR | NR |
| Abdominal Pain | 15.4% (2/13) | NR | NR | NR |
| Vomiting | 7.7% (1/13) | NR | NR | NR |
| Loss of Appetite | 69.2% (9/13) | 46.2% (6/13) | NR | 7.7% (1/13) |
| Fatigue | 92.3% (12/13) | 92.3% (12/13) | 69.2% (9/13) | 23.1% (3/13) |
| Myalgia | 53.8% (7/13) | 15.4% (2/13) | NR | NR |
| Jaundice | 15.4% (2/13) | 7.7% (1/13) | NR | NR |
|  | | | | |
| **AM-LUM** | **Before**  **Treatment (D0)** | **D1-24 h** | **D2-48 h** | **D3-72 h** |
| Rigors/Chills | 92.9% (13/14) | 7.1% (1/14) | 7.1% (1/14) | NR |
| Sweating | 92.9% (13/14) | 14.3% (2/14) | 7.1% (1/14) | NR |
| Headache | 100.0% (14/14) | 92.9%(13/14) | 50.0% (7/14) | 7.1% (1/14) |
| Nausea | 14.3% (2/14) | NR | NR | NR |
| Abdominal Pain | 14.3% (2/14) | NR | NR | NR |
| Vomiting | 14.3% (2/14) | NR | NR | NR |
| Loss of Appetite | 71.4% (10/14) | 42.9% (6/14) | 35.7% (5/14) | NR |
| Fatigue | 100.0% (14/14) | 100.0% (14/14) | 50.0% (7/14) | 14.3% (2/14) |
| Myalgia | 50.0% (7/14) | 28.6% (4/14) | NR | NR |
| Jaundice | 21.4% (3/14) | NR | NR | NR |

NR – Not reported by participant; AE recorded immediately before dosing at D0, D1, D2 and D3
