# Supplementary material for: Susceptibility of Plasmodium falciparum to artemisinins and Plasmodium vivax to chloroquine in Phuoc Chien Commune, Ninh Thuan Province, south-central Vietnam
Source: Malar J. 2019 Jan 17;18:10. doi: 10.1186/s12936-019-2640-2 (PMC6335800; doi:10.1186/s12936-019-2640-2)
Supplement: Supplementary file 4 — Additional file 4: Table S1. In vitro drug susceptibility data (IC50, nM) of the laboratory control Plasmodium falciparum D6 and MRA1240 lines and the 11 field isolates of Plasmodium falciparum collected from study participants. Table S2. Admission parasitaemia and parasite clearance time of the 11 adult patients who provided a blood sample for in vitro drug susceptibility testing (see Table S1 for IC50 values). Table S3. Determination of the proportion of viable parasites (% survival) in DHA-treated compared to untreated cultures by microscopy. Table S4. Determination of the proportion of viable parasites (% survival) in PPQ-treated compared to untreated cultures by microscopy. [file 12936_2019_2640_MOESM4_ESM.docx]

**Additional file 4:**

**Table S1.** In vitro drug susceptibility data (IC_50_, nM) of the laboratory control *Plasmodium falciparum* D6 and MRA1240 lines and the 11 field isolates of *Plasmodium falciparum* collected from study participants.

| **Drug** | **D6** | | | **MRA1240** | | |
| --- | --- | --- | --- | --- | --- | --- |
| **ATQ** | 0.1 | ± | 0.0 | 1.5 | ± | 0.4 |
| **CQ** | 13 | ± | 4 | 97 | ± | 21 |
| **dAQ** | 8.2 | ± | 0.3 | 26 | ± | 4 |
| **DHA** | 1.9 | ± | 0.1 | 1.0 | ± | 0.7 |
| **LUM** | 108 | ± | 12 | 139 | ± | 9 |
| **MQ** | 40 | ± | 1 | 84 | ± | 8 |
| **PPQ** | 23 | ± | 2 | 17 | ± | 5 |
| **PRN** | 5.5 | ± | 1.0 | 3.2 | ± | 2.0 |

| **Drug** | **VAS06** | | | **VAS07** | | | **VAS08** | | | **VAS09** | | | **VAS10** | | |
| --- | --- | --- | --- | --- | --- | --- | --- | --- | --- | --- | --- | --- | --- | --- | --- |
| **ATQ** | 0.17 | ± | 0.02 | 0.2 | ± | 0.0 | 0.19 | ± | 0.00 | 0.5 | ± | 0.4 | 0.2 | ± | 0.1 |
| **CQ** | 94 | ± | 28 | 42 | ± | 11 | 100 | ± | 50 | 67 | ± | 15 | 72 | ± | 9 |
| **dAQ** | 12.8 | ± | 0.4 | 9.4 | ± | 3.1 | 10.3 | ± | 0.5 | 21 | ± | 0 | 17 | ± | 5.8 |
| **DHA** | 0.95 | ± | 0.14 | 1.7 | ± | 0.1 | 1.0 | ± | 0.3 | 1.4 | ± | 0.5 | 0.5 | ± | 0.0 |
| **LUM** | 37 | ± | 6 | 43 | ± | 9 | 31 | ± | 2 | 45 | ± | 16 | 6 | ± | 1 |
| **MQ** | 19 | ± | 4 | 30 | ± | 0 | 22 | ± | 0.3 | 25 | ± | 1 | 4.6 | ± | 0.2 |
| **PPQ** | 13.5 | ± | 1.3 | 13.1 | ± | 0.9 | 12 | ± | 1.0 | 4.6 | ± | 0.9 | 5.4 | ± | 0.3 |
| **PRN** | 3.4 | ± | 0.2 | 3.7 | ± | 0.2 | 3.1 | ± | 0.6 | 5.4 | ± | 1.3 | 3.1 | ± | 0.4 |

| **Drug** | **VAL05** | | | **VAL06** | | | **VAL07** | | | **VAL08** | | | **VAL09** | | | **VAL10** | | |
| --- | --- | --- | --- | --- | --- | --- | --- | --- | --- | --- | --- | --- | --- | --- | --- | --- | --- | --- |
| **ATQ** | 0.2 | ± | 0.1 | 0.6 | ± | 0.1 | 0.17 | ± | 0.02 | 0.2 | ± | 0.0 | 0.2 | ± | 0.1 | 0.2 | ± | 0.1 |
| **CQ** | 54 | ± | 7 | 8.5 | ± | 0.7 | 51 | ± | 19 | 33 | ± | 4 | 20 | ± | 5 | 16 | ± | 6 |
| **dAQ** | 10.9 | ± | 0.3 | 5.8 | ± | 0.4 | 11.2 | ± | 0.3 | 4.1 | ± | 2.5 | 13.8 | ± | 1.8 | 13.3 | ± | 4 |
| **DHA** | 0.6 | ± | 0.1 | 1.9 | ± | 0.2 | 1.0 | ± | 0.4 | 0.8 | ± | 0.0 | 1.2 | ± | 0.0 | 1.0 | ± | 0.3 |
| **LUM** | 16.3 | ± | 4.2 | 53.1 | ± | 18.2 | 35 | ± | 9 | 35 | ± | 19 | 25 | ± | 4 | 25 | ± | 0 |
| **MQ** | 10.2 | ± | 1.5 | 30.6 | ± | 3.5 | 24 | ± | 1 | 14.2 | ± | 3.7 | 11.2 | ± | 2.1 | 7.2 | ± | 1.8 |
| **PPQ** | 11.7 | ± | 1.2 | 14.5 | ± | 1.0 | 10.3 | ± | 2.6 | 8.4 | ± | 1.5 | 8.9 | ± | 1.1 | 7.7 | ± | 0.2 |
| **PRN** | 1.8 | ± | 0.3 | 3.3 | ± | 0.3 | 2.9 | ± | 0.2 | 2.0 | ± | 0.5 | 3.5 | ± | 0.2 | 4.6 | ± | 1.1 |

**Table S2**. Admission parasitaemia and parasite clearance time of the 11 adult patients who provided a blood sample for in vitro drug susceptibility testing (see Table S1 for IC_50_ values).

| **Drug** | **Patient ID** | **Admission Parasitaemia (parasites/µL)** | **Parasite Clearance Time (h)** |
| --- | --- | --- | --- |
| AS+DHA-PPQ | VAS06A | 91,361 | 48 |
| AS+DHA-PPQ | VAS07A | 6,061 | 36 |
| AS+DHA-PPQ | VAS08A | 4,536 | 24 |
| AS+DHA-PPQ | VAS09A | 87,131 | 12 |
| AS+DHA-PPQ | VAS10A | 23,549 | 36 |
|  |  |  |  |
| AM-LUM | VAL05A | 34,030 | 36 |
| AM-LUM | VAL06A | 2,958 | 36 |
| AM-LUM | VAL07A | 26,921 | 36 |
| AM-LUM | VAL08A | 27,393 | 36 |
| AM-LUM | VAL09A | 12,550 | 60 |
| AM-LUM | VAL10A | 2,078 | 24 |

**Table S3.** Determination of the proportion of viable parasites (% survival) in DHA-treated compared to untreated cultures by microscopy.

| **Strain** | **P (%)**  **at 0 h** | **P (%)**  **at 72 h** | **Growth Rate**  **(NE/INI)** | **% Live parasite**  **at 72 h** | **(DHA/NE)*100%**  **at 72 h (%)** |
| --- | --- | --- | --- | --- | --- |
| MRA1239^S^ | 1.00 | 6.35 | 6.35 | 0.01 | 0.16 |
| MRA1240^R^ | 1.00 | 7.30 | 7.30 | 0.53 | 7.26 |
| VAS06 | 1.10 | 4.60 | 4.18 | 0.05 | 1.20 |
| VAS07 | 1.10 | 4.40 | 4.00 | 0.06 | 1.50 |
| VAS08 | 1.00 | 5.60 | 5.60 | 0.04 | 0.71 |
| VAS09 | 0.90 | 6.00 | 6.67 | 0.06 | 0.90 |
| VAS10 | 0.70 | 4.25 | 6.07 | 0.06 | 0.99 |

P – parasitaemia; INI - initial parasitaemia; NE - non‐exposed; DHA – DHA treated

**Table S4.** Determination of the proportion of viable parasites (% survival) in PPQ-treated compared to untreated cultures by microscopy.

| **Strain** | **P (%)**  **at 0 h** | **P (%)**  **at 72 h** | **Growth Rate (NE/INI)** | **% Live parasite**  **at 72 h** | **(PPQ/NE)*100%**  **at 72 h (%)** |
| --- | --- | --- | --- | --- | --- |
| MRA1239^S^ | 1.00 | 5.20 | 5.20 | 0.01 | 0.19 |
| MRA1240^R^ | 1.00 | 6.80 | 6.80 | 0.14 | 2.06 |
| VAS06 | 1.10 | 3.06 | 2.78 | 0.05 | 1.80 |
| VAS07 | 1.10 | 3.30 | 3.00 | 0.12 | 4.00 |
| VAS08 | 1.00 | 4.20 | 4.20 | 0.07 | 1.67 |
| VAS09 | 0.90 | 5.40 | 6.00 | 0.02 | 0.33 |
| VAS10 | 0.70 | 3.60 | 5.14 | 0.04 | 0.78 |

P – parasitaemia; INI - initial parasitaemia; NE - non‐exposed; PPQ- PPQ treated
