# Supplementary material for: Susceptibility of Plasmodium falciparum to artemisinins and Plasmodium vivax to chloroquine in Phuoc Chien Commune, Ninh Thuan Province, south-central Vietnam
Source: Malar J. 2019 Jan 17;18:10. doi: 10.1186/s12936-019-2640-2 (PMC6335800; doi:10.1186/s12936-019-2640-2)
Supplement: Supplementary file 5 — Additional file 5: Table S1. Adverse events before and after treatment of Vietnamese patients with CQ for uncomplicated vivax malaria. [file 12936_2019_2640_MOESM5_ESM.docx]

**Additional file 5: Table S1.** Adverse events before and after treatment of Vietnamese patients with CQ for uncomplicated vivax malaria

| **CQ** | **Before**  **Treatment (D0)** | **D1-24 h** | **D2-48 h** | **D3-72 h** |
| --- | --- | --- | --- | --- |
| Rigors/Chills | 93.8% (15/16) | 12.5% (2/16) | 18.8% (3/16) | NR |
| Sweating | 93.8% (15/16) | 18.8% (3/16) | 6.3% (1/16) | NR |
| Headache | 100.0% (16/16) | 87.5%(14/16) | 37.5% (6/16) | 12.5% (2/16) |
| Nausea | 18.8% (3/16) | NR | NR | NR |
| Abdominal Pain | 6.3% (1/16) | NR | NR | NR |
| Vomiting | 12.5% (2/16) | NR | NR | NR |
| Loss of Appetite | 68.8% (11/16) | 50.0% (8/16) | 18.8% (3/16) | NR |
| Fatigue | 93.8% (15/16) | 100.0% (16/16) | 75.0% (12/16) | 31.3% (5/16) |
| Myalgia | 31.3% (5/16) | 12.5% (2/16) | 12.5% (2/16) | NR |
| Jaundice | 12.5% (2/16) | 6.3% (1/16) | NR | NR |

NR – Not reported by participant; AE recorded immediately before dosing at D0, D1, D2 and D3
